# Molecular profiling of low grade serous ovarian tumours identifies novel candidate driver genes

**DOI:** 10.18632/oncotarget.5438

**Published:** 2015-10-19

**Authors:** Sally M. Hunter, Michael S. Anglesio, Georgina L. Ryland, Raghwa Sharma, Yoke-Eng Chiew, Simone M. Rowley, Maria A. Doyle, Jason Li, C. Blake Gilks, Phillip Moss, Prue E. Allan, Andrew N. Stephens, David G. Huntsman, Anna deFazio, David D. Bowtell, Australian Ovarian Cancer Study Group, Kylie L. Gorringe, Ian G. Campbell

**Affiliations:** ^1^ Centre for Cancer Genomics and Predictive Medicine, Peter MacCallum Cancer Centre, East Melbourne, Australia; ^2^ The Department of Pathology, University of Melbourne, Parkville, Australia; ^3^ The Sir Peter MacCallum Department of Oncology, University of Melbourne, Parkville, Australia; ^4^ Department of Pathology and Laboratory Medicine, University of British Columbia, Vancouver, Canada; ^5^ Anatomical Pathology, University of Sydney and University of Western Sydney at Westmead Hospital, Westmead, Australia; ^6^ Department of Gynaecological Oncology, Westmead Hospital, Westmead, Australia; ^7^ Centre for Cancer Research, University of Sydney at Westmead Millennium Institute, Westmead Hospital, Westmead, Australia; ^8^ Bioinformatics Core Facility, Peter MacCallum Cancer Centre, East Melbourne, Victoria, Australia; ^9^ Genetic Pathology Evaluation Centre, Vancouver General Hospital, Vancouver, Canada; ^10^ Anatomical Pathology, Peter MacCallum Cancer Centre, East Melbourne, Australia; ^11^ Centre for Cancer Research, MIMR-PHI Institute of Medical Research, Clayton, Victoria, Australia; ^12^ Department of Molecular and Translational Sciences, Monash University, Clayton, Victoria, Australia; ^13^ Epworth Research Institute, Epworth HealthCare, Richmond, Victoria, Australia; ^14^ The full Australian Ovarian Cancer Study Group can be found at http://www.aocstudy.org; ^15^ Current address: Saint John Regional Hospital, NB, Canada

**Keywords:** exome, borderline, serous ovarian tumor, genomics, copy number

## Abstract

Low grade serous ovarian tumours are a rare and under-characterised histological subtype of epithelial ovarian tumours, with little known of the molecular drivers and facilitators of tumorigenesis beyond classic oncogenic *RAS/RAF* mutations. With a move towards targeted therapies due to the chemoresistant nature of this subtype, it is pertinent to more fully characterise the genetic events driving this tumour type, some of which may influence response to therapy and/or development of drug resistance. We performed genome-wide high-resolution genomic copy number analysis (Affymetrix SNP6.0) and mutation hotspot screening (*KRAS, BRAF, NRAS, HRAS, ERBB2* and *TP53)* to compare a large cohort of ovarian serous borderline tumours (SBTs, *n* = 57) with low grade serous carcinomas (LGSCs, *n* = 19). Whole exome sequencing was performed for 13 SBTs, nine LGSCs and one mixed low/high grade carcinoma. Copy number aberrations were detected in 61% (35/57) of SBTs, compared to 100% (19/19) of LGSCs. Oncogenic *RAS/RAF/ERBB2* mutations were detected in 82.5% (47/57) of SBTs compared to 63% (12/19) of LGSCs, with *NRAS* mutations detected only in LGSC. Some copy number aberrations appeared to be enriched in LGSC, most significantly loss of 9p and homozygous deletions of the *CDKN2A/2B* locus. Exome sequencing identified *BRAF, KRAS, NRAS, USP9X* and *EIF1AX* as the most frequently mutated genes. We have identified markers of progression from borderline to LGSC and novel drivers of LGSC. USP9X and EIF1AX have both been linked to regulation of mTOR, suggesting that mTOR inhibitors may be a key companion treatment for targeted therapy trials of MEK and RAF inhibitors.

## INTRODUCTION

Low grade serous ovarian carcinomas (LGSCs) are an under-characterised histological subtype of epithelial ovarian tumours. Although LGSC follow a relatively indolent clinical course, they occur at a younger age than high grade serous carcinomas (HGSC) and patients with higher stage disease typically have poor overall survival due to the inherent chemo-resistant nature of LGSCs [1]. With *a* < 5% response rate to chemotherapy [2], the clinical behaviour of these tumours is in stark contrast to the 80% response rate to primary chemotherapy observed for women with HGSC.

Serous borderline tumours (SBTs) are considered to be non-invasive precursor lesions to LGSC and a significant proportion, particularly late stage, will recur and progress to carcinoma given sufficient clinical follow-up [3]. Some histological features have been associated with a greater likelihood of progression and recurrence, such as invasive implants and micropapillary growth pattern [4]. It is currently unknown whether these histological patterns are associated with specific molecular events; if so, molecular profiling of borderline tumours could offer significant improvements to predicting the likelihood of progression and recurrence.

Little is known of the molecular drivers and facilitators of tumourigenesis in SBT or LGSC beyond the classic oncogenic *KRAS* and *BRAF* mutations, and more recently *ERBB2* and *NRAS* mutations [5, 6]. Approximately 40–60% of LGSC are *RAS*/*RAF* mutation positive [6, 7], leaving a significant proportion with unidentified drivers. In addition, *RAS*/*RAF* mutations alone cannot explain the progression of SBT to carcinoma as 70–80% of SBTs already carry oncogenic *KRAS, BRAF* and *ERBB2* mutations [5]. Thus, the molecular events underlying the transition to carcinoma remain undetermined.

To identify novel molecular drivers and molecular features that might be predictive of clinical behaviour we performed genome-wide high-resolution copy number analysis on a large cohort of SBTs and LGSCs, as well as exome sequencing of a subset of cases. We identified recurrent oncogenic mutations and copy number aberrations (CNAs) in the SBTs that were significantly associated with clinical features, and CNAs and mutations significantly associated with carcinomas that therefore potentially underlie tumour progression. Through exome sequencing we also identified somatic mutations in a number of genes that have not been previously associated with LGSC. With the current trend towards targeted therapies, in particular MEK and RAF inhibitor trials for LGSC, there is an imperative to understanding the molecular events underlying LGSC development as well as those that may influence treatment outcome and resistance mechanisms.

## RESULTS

### RAS/RAF pathway mutations in SBT and LGSC

Mutation screening was performed on 57 SBT and 19 LGSC (Table 1) by Sanger sequencing at known mutational hotspots in the genes *KRAS, BRAF, NRAS, HRAS, ERBB2*, and exons 5–8 of *TP53* (Table 2, Supplementary Tables S1, S2). Overall, RAS/RAF pathway mutations were identified in 82.5% of SBT cases and 63% of LGSCs, similar to previous reports [5]. Of note, two SBTs were *KRAS-BRAF* double mutants (Supplementary Table S1), with classic *KRAS*^G12^ mutations (p.G12D and p.G12V) and concurrent kinase activating *BRAF* mutations (p.G464V and p.G469A) that occur in the highly conserved P-loop. Frequent overlap of *BRAF* P-loop mutations with *KRAS* mutations has been previously reported in colorectal carcinomas [8].

**Table 1:** SBT and LGSC cohort clinical features

Feature	Property	SBT (*n* = 57)	LGSC (*n* = 19)
Median age (years)		49 (range 22–80)	62 (range 23–83)
Laterality	Right ovary	24 (44%)	2 (11%)
	Left ovary	9 (16%)	1 (5.5%)
	Unspecified, unilateral	2 (3.5%)	1 (5.5%)
	Bilateral	22 (40%)	14 (74%)
	Not known		1 (5.5%)
Stage	I	17 (50%)	1 (5.5%)
	II	4 (12%)	0
	III	12 (35%)	13 (72%)^a^
	IV	1 (3%)	1 (5.5%)
	Not known		4 (21%)
Microinvasion		9^b^ (15.8%)	NA
Implants		18^c^ (32%)	NA
Micropapillary pattern		0 (0%)	4 (21%)

a Staging information for *n* = 7 stage 3 carcinomas was derived from the pathology report and represents the minimum possible stage i.e. these cases are at least stage 3;

b Total based on pathology review performed as part of this study;

c Total based on pathology report. NA, not applicable.

**Table 2:** Comparison of gene mutation frequencies in serous ovarian tumours

Mutation	SBT (*n* = 57)	LGSC (*n* = 19)	Fisher's exact test *p*-value	HGSC(TCGA, *n* = 316)	HGSC (non-TCGA, *n* = 40)
*KRAS*	21 (36.8%)	4 (21%)	0.2657	2 (0.6%)	2 (5%)
*BRAF* (V600E)	22 (38.6%)	3 (16%)	0.0920	0	0
*BRAF* (non-V600E)	2 (3.5%)	0	1.0000	2 (0.6%)	0
*HRAS*	1 (1.8%)	0	1.0000	0	0
***NRAS***	**0**	**5 (26%)**	**0.0006**	2 (0.6%)	1 (2.5%)
*ERBB2 (exon 20)*	3 (5.3%)	0	0.5686	1 (0.3%)	NA
*TP53*	0	0	-	300^a^ (95%)	36 (90%)
WT	10 (17.5%)	8 (42%)	0.0577	16 (5%)	5 (10%)

a Total includes cases with homozygous deletion of *TP53* locus. NA, Not Assessed.

Interestingly, activating p.Q61 *NRAS* mutations were identified in 26% of the LGSCs but were absent among 57 SBTs (*p* = 0.0006, Emmanuel *et al*. [6] and this study) (Table 2). The mutation rate of other oncogenes was not significantly different between the LGSCs and SBTs; however there was a trend towards a lower frequency of *BRAF* and *KRAS* mutants in the LGSCs and a higher rate of “wildtype” tumours, where no oncogenic mutation was identified (Table 2). No *TP53* mutations were identified in any SBT or LGSC cases.

### Copy number aberrations are associated with progression of SBT to LGSC but are less frequent compared to HGSC

Genomic copy number aberrations (CNAs and copy neutral loss of heterozygosity (CNLOH)) were identified in the epithelial component of 61.4% of SBTs (Supplementary Table S1), and 100% of LGSCs (Supplementary Table S2), similar to the frequencies in previous reports [9, 10]. The most frequent CNAs observed across SBTs and LGSCs involved loss of 1p (20%), 9p (14%) and 19 (11%), and gain of 7/7q (18%), 8/8q (20%) and 12/12p (17%).

**Figure 1:**
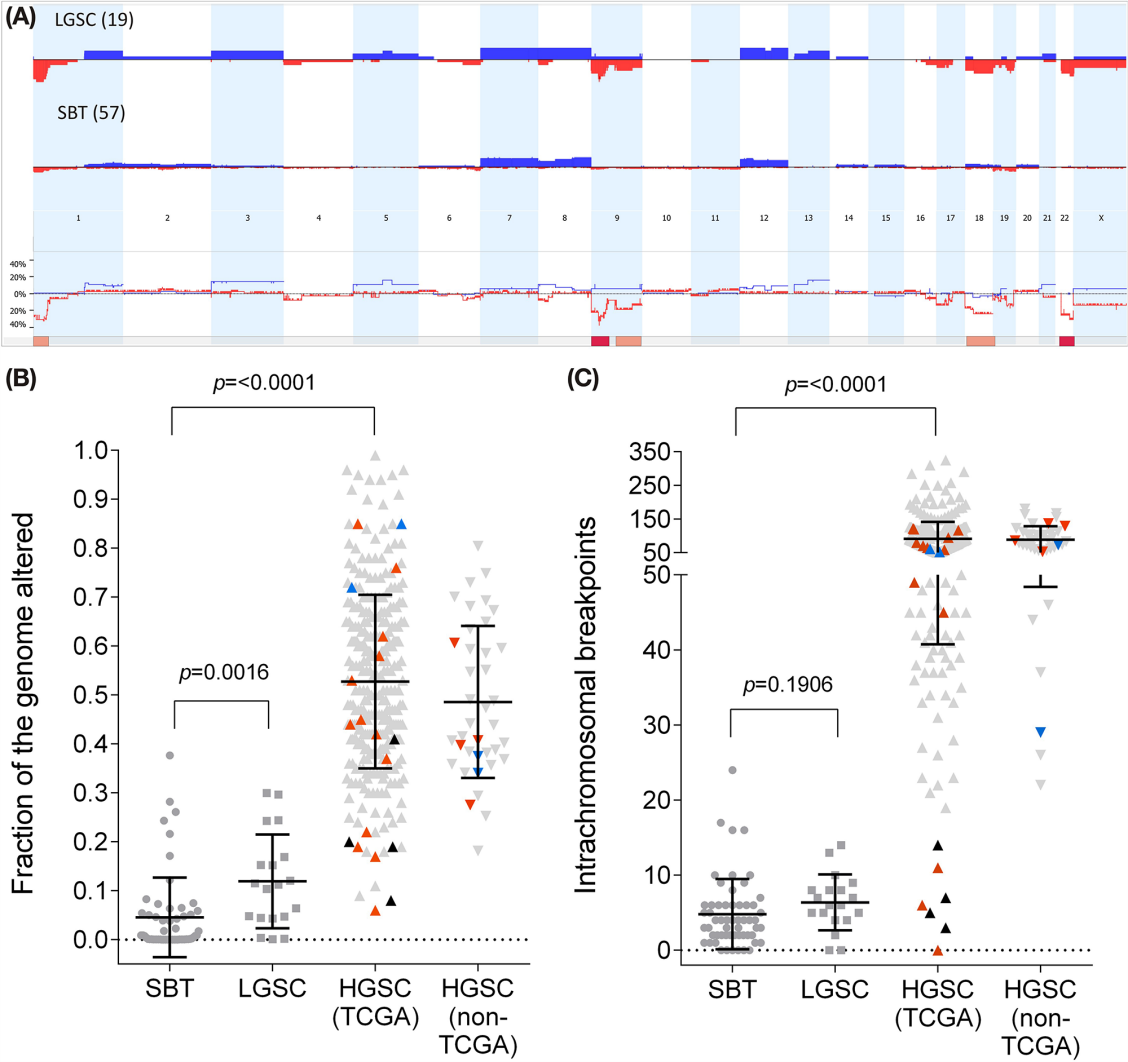
Genomic aberration levels in serous ovarian tumours **A.** Cumulative copy number aberrations of LGSC cohort (*n* = 19, top panel) and SBT (*n* = 57, middle panel); and copy number difference plot (lower panel). Blue indicates copy number gains while red indicates copy number losses. The solid pink bars underneath indicate regions of genomic aberration that are enriched in LGSC compared to SBT (*p* < 0.01, FET), while solid red bars indicate highly significant enrichment (*p* < 0.001, FET). **B.** Fraction of the genome altered and **C.** Intrachromosomal breakpoint counts. Data points represent individual tumours, with mean and standard deviation of each cohort plotted. For the HGSC cohorts, light grey indicates typical *TP53* mutant tumour, orange indicates *TP53* wildtype tumour, blue indicates *RAS/RAF* mutations co-occuring with *TP53* mutations, black indicates *RAS/RAF* mutant-*TP53* wildtype tumour.

Significant enrichment of specific CNAs was observed in the LGSCs compared to the SBTs (Figure 1A, Supplementary Table S3). The most statistically significant event involved loss/LOH (loss of heterozygosity) of 9p (2% of SBTs (1/57) versus 53% of LGSCs (10/19), *p* < 0.0001, Supplementary Table S3). Sequencing of the *CDKN2A* locus did not identify any mutations in the LGSCs, however the single SBT with 9p LOH (IC508) was found to have a 19 bp deletion that is predicted to result in truncation of both p16^INK4A^ and p19^ARF^ (Supplementary Table S1). Three of the LGSCs with LOH also harboured homozygous deletions targeting the *CDKN2A/2B* locus at 9p21.3. We further evaluated p16 through immunohistochemical staining (Supplementary Figures S1, S2). There was a trend towards weaker staining in the LGSCs (*n* = 25) compared to the SBTs (*n* = 30) (*p* = 0.12, Fisher's Exact Test, FET), while HGSCs (*n* = 192) had a higher proportion of strongly staining cases (*p* < 0.0001, FET) (Supplementary Table S4). A trend towards weaker staining associated with LOH of 9p in SBT and LGSC (*p* = 0.07, FET) was also observed (Supplementary Table S5, Supplementary Figures S1 and S2).

Loss of chromosome 22 or gain of chromosome 13, was not observed in any of the SBT cohort, but these aberrations were observed in 32% and 16% of the LGSCs, respectively (Figure 1A, Supplementary Table S3). Losses of 1p, 9q, 18q and X were aberrations observed in the SBTs that was significantly enriched in the LGSCs (Figure 1, Supplementary Table S3). The most frequent copy number gains observed in the SBT cohort (chromosomes 7, 8 and 12) were not significantly enriched in the LGSC cases.

The LGSCs in this cohort were found to have, on average, a greater fraction of their genomes altered (FGA) compared to the SBT cohort (*p* = 0.0001, Mann-Whitney test; Figure 1B), however, a subset of the SBT cohort had equivalent FGA to the LGSCs. LGSCs also had more intrachromosomal breakpoints (*p* = 0.03, Mann-Whitney-test; Figure 1C). The SBTs and LGSCs were compared to a mixed cohort of grade 2–3 serous carcinomas, including seven unique to this study, 33 previously published [11] and 316 from TCGA [12]. Comparison of rates of intrachromosomal breakpoints and FGA revealed a broad range of genomic aberration in all HGSC cohorts compared to the generally low rates in SBTs and LGSCs (Figure 1B–1C). Considering FGA and number of intrachromosomal breakpoints in combination, as expected the SBT and LGSC cohorts clustered together with low genomic aberration levels (Supplementary Figure S3). HGSCs displayed a wide range of breakpoint rates and FGA, but tended not to cluster with the SBTs and LGSCs.

Seven reported HGSCs appeared to have a low-grade pattern of genomic aberrations and clustered with the SBT/LGSC (<18 breakpoints, Supplementary Figure S3). These cases were enriched for *RAS/RAF* mutations (4/7 had a *RAS/RAF* mutation compared to 4/348, *p* < 0.0001, FET) and had no detectable *TP53* mutations. These seven low grade-like tumours, part of the 2011 TCGA publication [12] were the centre of a recent study [13] suggesting they have wildtype TP53 and least four are likely to be SBT (*n* = 1) and LGSC (*n* = 3). The remainder were reported by Wong *et al.* [13] as malignant mixed Müllerian tumour (*n* = 1) and HGSC (*n* = 2). In contrast, four HGSC cases with *RAS/RAF* mutations and > 18 breakpoints all had a *TP53* mutation (Figure 1C, Supplementary Figures S3 and S4).

### RAS/RAF pathway aberrations differ in their association with molecular and clinical features

We evaluated whether the global genomic profile of a tumour was associated with particular oncogenic mutations. A substantial proportion of the SBT cohort (22/57 cases, Figure 1A, Supplementary Table S6) did not harbour any detectable somatic CNAs or CNLOH. There was a trend for these cases to have either a *BRAF* mutation (13/24, 58%, *p* = 0.1744, FET) or an *ERBB2* mutation (3/3, *p* = 0.0526, FET; Supplementary Table S6), but this did not reach statistical significance. Considering mutual exclusivity between chromosomal instability and microsatellite instability (MSI) [14], reports of overlap between *BRAF*^V600E^ mutations with MSI in colorectal tumours [15] and reports of MSI in SBTs [16], 14 tumours with *BRAF*^V600E^ mutations were tested for MSI. All 14 tumours tested were found to be microsatellite stable (Supplementary Table S1). However, all three *BRAF* mutant LGSCs identified in this cohort had CNAs (Supplementary Table S2), perhaps suggesting a requirement for copy number events for progression in a *BRAF*-mutant context. In contrast to *BRAF* and *ERBB2* mutations, activating *KRAS* mutations were significantly associated with the presence of genomic aberrations in SBT (*p* = 0.0199, FET; Supplementary Table S6) and a larger FGA (Supplementary Figure S5, *p* = 0.04, two-tailed *t*-test).

We then determined whether clinical features were associated with oncogenic mutations. For SBTs, bilaterality was found to be significantly more common for *KRAS* mutant tumours (63.2% versus 26.5%, *p* = 0.0101, FET; Supplementary Table S6), whereas the majority of *BRAF*^V600E^ mutant tumours (76.2%) were unilateral. The presence of a *KRAS* mutation was also significantly associated with a tumour stage > I (*p* = 0.0240, FET; Supplementary Table S6). No statistically significant associations were identified between micropapillary changes or microinvasion and a single oncogenic mutation or specific CNA. Intriguingly however, both *BRAF-KRAS* double-mutant tumours had reported micropapillary architecture (compared to 5/55 cases of remaining cohort, *p* = 0.01, FET).

Associating clinical features with oncogenic mutations or CNAs in the LGSC cohort was difficult due to the majority of these tumours being bilateral and late stage (Table 1). The presence of reported micropapillary growth pattern was not enriched in LGSCs (3/19) compared to SBTs (7/57) (*p* = 0.7040, FET), however, based on pathology review of microdissected areas 4/19 LGSCs displayed a dominant micropapillary growth pattern compared to 0/57 SBTs (*p* = 0.003, FET). Micropapillary architecture appeared to be associated with the presence of a *NRAS*^Q61^ mutation in these LGSCs (3/4 of cases), however the numbers are small.

### Exome sequencing identifies novel drivers in LGSC

A substantial proportion of SBTs (17.5%) and LGSCs (42%) did not have oncogenic mutations in the RAS/RAF pathway. Additionally, it is likely that ancillary events alongside *RAS/RAF* mutations are required to initiate and drive neoplasia and these are essentially unknown. In order to address these knowledge gaps we undertook exome sequencing of 13 SBTs (including three *RAS*/*RAF* mutation wildtype), nine LGSCs (including three *RAS*/*RAF* mutation wildtype) and one tumour with primarily low grade histology but foci of grade 2/3 differentiation.

**Figure 2:**
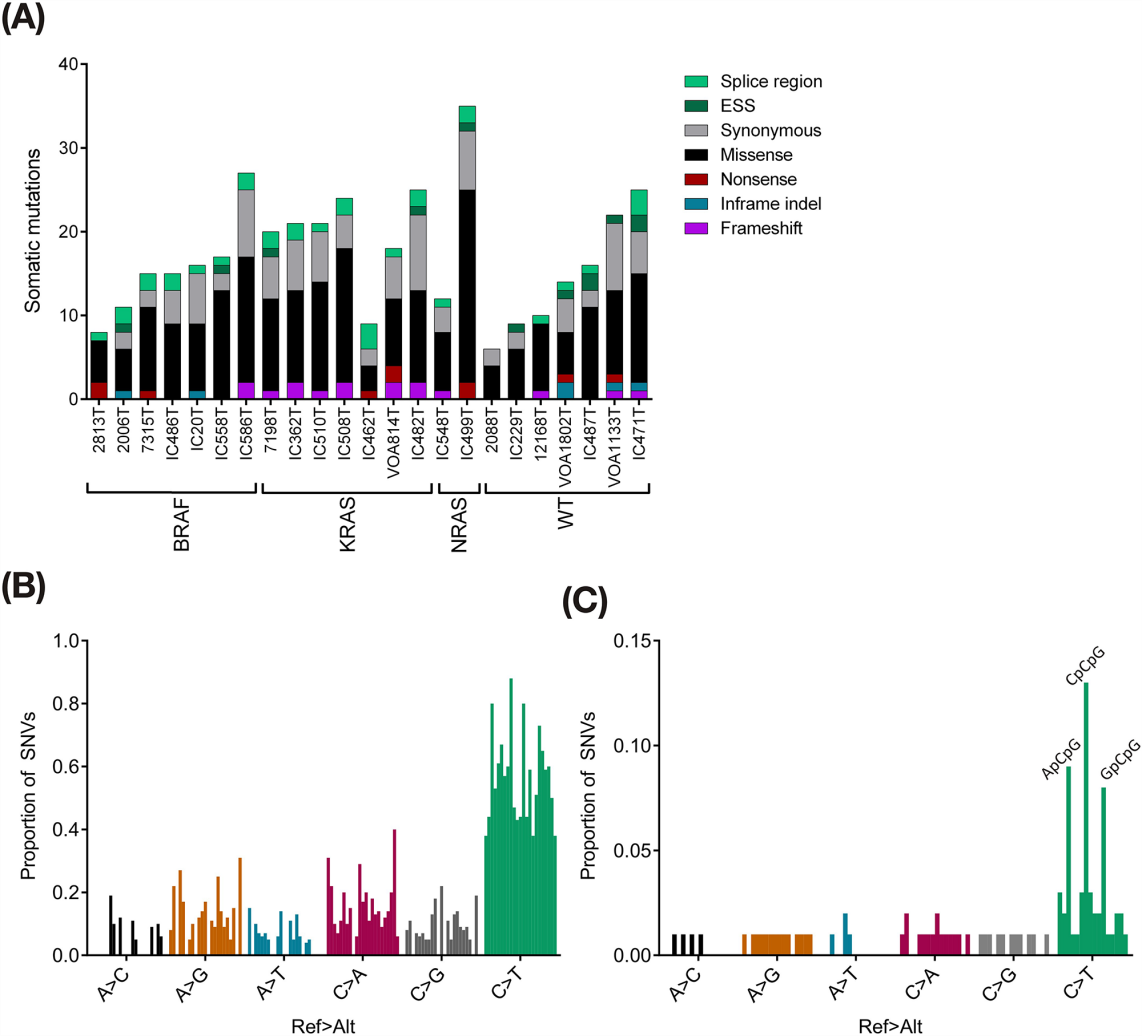
Exome sequencing summary **A.** Exome variant counts, by consequence type. **B.** Tumour-level SNV counts demonstrating an enrichment of the common C > T change, which is consistent with age-acquired cytosine deamination. **C.** Aggregate mutation signature. Peaks at NpCpG > NpTpG, with the exception of TpCpG > TpTpG.

We identified a total of 396 somatic variants in the 23 tumours using stringent filtering, and an additional 19 somatic variants identified by Pindel only. We undertook Sanger validation of 106/396 stringent variants, achieving a validation rate of 91%, and 10/19 Pindel-only variants, achieving a validation rate of 50% (Supplementary Table S7). Mutation rates in SBT and LGSC cohorts were found to be relatively low, but quite similar with ranges of 6–24 (median 15) and 9–27 (median 18) mutations, respectively (Figure 2A). These rates are similar to those previously reported in two small studies of SBT and LGSC [17, 18], and 4–5 times lower than the average 1.8 mutations/Mb reported for HGSC [12]. The carcinoma with mixed low and high grade histology had the highest mutation rate with 35 SNVs and small indels detected. Although the low mutation rate limits the power of mutation signature analyses, there was a clear trend towards C > T/G > A transitions across the tumour cohort (Figure 2B), with an enrichment in the context of NpCpG, with the exception of TpCpG (Figure 2C).

**Figure 3:**
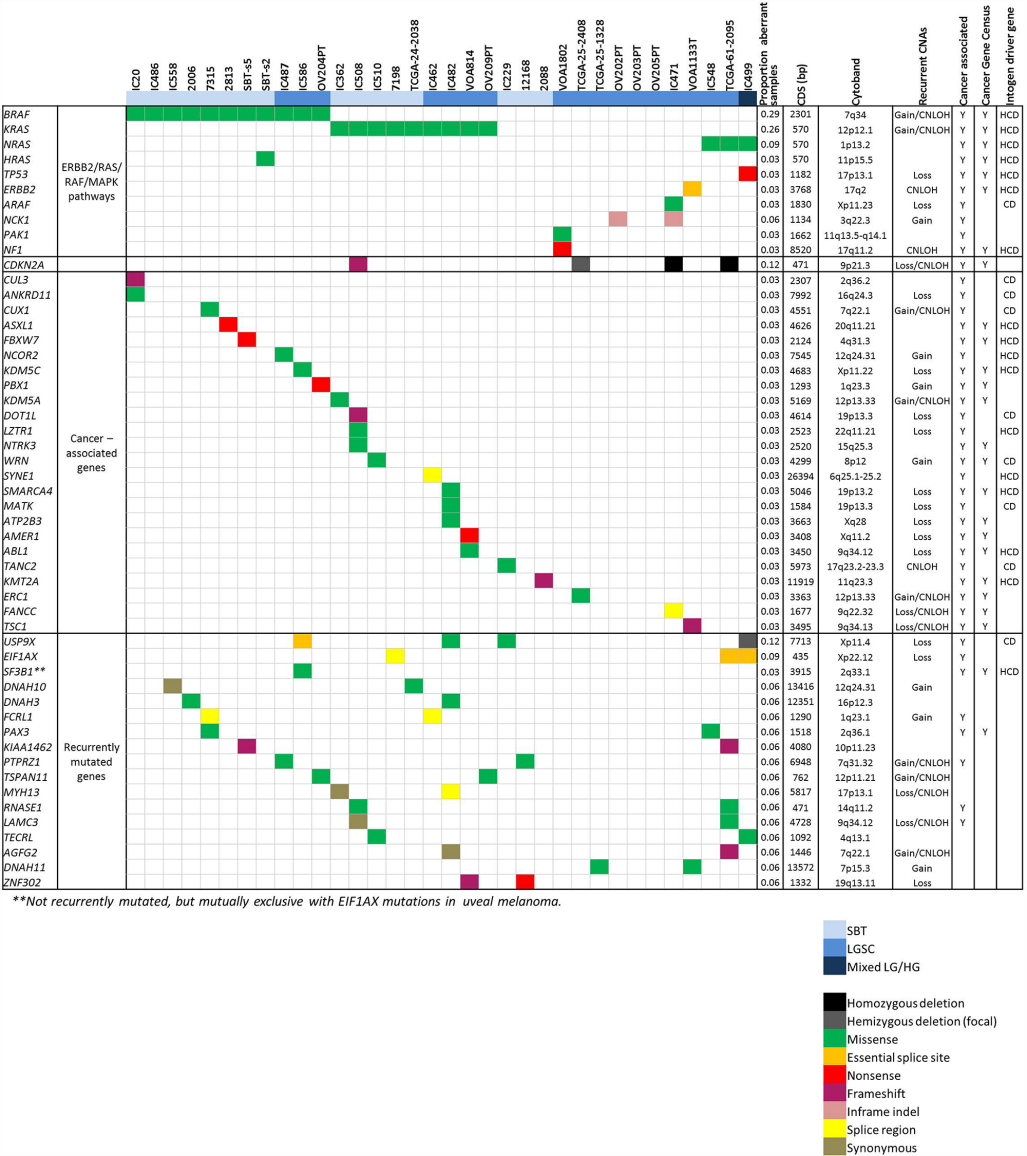
Mutation patterns in low grade serous ovarian tumours Genes recurrently mutated and cancer genes mutated in low grade serous ovarian tumours. Cancer association is based on literature; COSMIC Cancer Gene Census (accessed January 2015); IntOGen driver genes are classified as HCD, high confidence drivers or CD, candidate drivers.

Three of six “wildtype” tumours had identifiable alternative RAS/MAPK or ERBB2 pathway mutations–novel *ARAF* (p.(G11E)) and *NCK1* (p.(E336del)) mutations in combination, *PAK1* (p.(S57_I58insFFR)) and *NF1* (p.(E1119X)) mutations in combination, and an *ERBB2* essential acceptor splice site mutation that results in skipping of exon 16 (Supplementary Figure S6A), which encodes part of the growth factor receptor IV domain, in combination with a truncating *TSC1* mutation (p.(P141fs11X)). We also identified 17 novel recurrently mutated genes (Figure 3). To increase the power of our analysis to detect significant genes outside of the RAS/RAF pathway, we analysed our data together with validated variants from the published studies of Jones *et al*. (*n* = 5 pure LGSC), Boyd *et al*. (*n* = 2 SBT) and TCGA cases reported by Wong *et al*. [13] as SBT (*n* = 1) or LGSC (*n* = 3); the results from the 34 tumours are summarised in Figure 3. A number of the recurrently mutated genes have been previously associated with tumourigenesis (*EIF1AX, USP9X, NCK1, RNASE1*), while other genes (*DNAH3, DNAH10, DNA11*) have no clear role in tumourigenesis and are potentially recurrently mutated by chance because of their large size. However, *DNAH3* mutations have been previously reported as cooperative with mutant APC in colon epithelial cell transformation [19] and cannot be completely discounted.

Mutation screening was performed for the genes *EIF1AX, SF3B1, USP9X, NCK1* and *RNASE1* using Sanger sequencing in a validation cohort of SBT (*n* = 44, *EIF1AX, n* = 23 *USP9X*) and 10 LGSC (*EIF1AX, SF3B1, USP9X, NCK1, RNASE1*). No additional mutations in 10 LGSC were identified in *RNASE1, NCK1* or *SF3B1*, bringing the aggregate frequency (including published exomes) of mutations in these genes to 7% (2/27), 7% (2/27) and 4% (1/27). Sanger sequencing of exons 1 and 2, including exon-intron boundaries, of *EIF1AX* in a validation cohort of 44 SBT and 10 LGSC identified an additional three somatic *EIF1AX* mutations in three LGSCs (Table 3), bringing the overall frequency to 4/27 (15%) LGSCs and 1/60 (1.7%) SBTs. Additionally, a rare *EIF1AX* 5′ UTR SNP (rs201653081) was identified in a single SBT (IC20T). All 44 coding exons of *USP9X* were screened in an additional 10 LGSC and 23 SBT and identified a nonsense mutation in one LGSC. The aggregate frequency of mutations in *USP9X* was 11% (3/27) LGSCs and 2.6% (1/39) SBTs (Table 3). All splicing variants in *EIF1AX* and *USP9X* were predicted to disrupt the coding sequence by Human Splicing Finder (HSF v2.4.1).

**Table 3:** *EIF1AX, USP9X* and *SF3B1* mutation screening

Sample	Histology	Gene	Mutation	*SIFT*	*Poly-Phen2*	*Condel*	*Human Splicing Finder*	X chromosome CN^d^ status	*RAS/RAF* mutation
IC325T	LGSC	***EIF1AX***	c.9G > T, p.(K3N)	*damaging* (0)	*benign* (0.419)	*deleterious* (0.660)	-	Neutral	*NRAS* p.Q61R
P4085T	LGSC	***EIF1AX***	c.4C > T, p.(P2S)^b^	*damaging* (0)	*benign* (0.000)	*deleterious* (0.597)	-	CN loss	*NRAS* p.Q61R
PHI679-07T	LGSC	***EIF1AX***	c.25G > C, p.(G9R)	*damaging* (0.03)	*prob_damaging* (0.996)	*deleterious* (0.641)	-	CN loss	*NRAS* p.Q61R
TCGA-61-2095 [12]	LGSC	***EIF1AX***	c.17-2A > C	-	-	-	*Predicted to reduce exon 2 by 6 bp*	Neutral	*NRAS* p.Q61R
7198T	SBT	***EIF1AX***	c.17-7G > C	-	-	-	*Predicted and validated to extend exon 2 by 6 bp*^c^	Neutral	*KRAS* p.G12V
IC499T	Mixed^a^	***EIF1AX***	c.17-2A > G	-	-	-	*Predicted and validated to reduce exon 2 by 6 bp* [27]	Neutral	*NRAS* p.Q61R
IC586T	LGSC	***SF3B1***	c.2098A > G, p.(K700E)-	*damaging* (0.01)	*prob_damaging* (0.993)	*deleterious* (0.856)	-	CN loss	*BRAF* p.V600E
IC586T	LGSC	***USP9X***	c.6563-6565+ 1delTAGG	-	-	-	*Predicted to extend exon 38 by 1 bp*^c^	CN loss	*BRAF* p.V600E
IC482T	LGSC	***USP9X***	c.6050T > G, p.(L2017R)	*damaging* (0)	*benign* (0.362)	*deleterious* (0.559)	-	Neutral	*KRAS* p.G12R; p.G12V
PHI679-07T	LGSC	***USP9X***	c.1222C > T, p.(Q408X)	-	-	-	-	CN loss	*NRAS* p.Q61R
IC229T	SBT	***USP9X***	c.5063A > T, p.(E1688V)	*damaging* (0)	*prob_damaging* (0.978)	*deleterious* (0.865)	-	Neutral	WT
IC499T	Mixed^a^	***USP9X***	-	-	-	-	-	Focal deletion encompassing majority of *USP9X*	*NRAS* p.Q61R

a Mixed histology–primarily grade 1 with foci of grade 2/3.

b No germline DNA available to confirm somatic status.

c see Supplementary Figure S6B.

d CN loss, copy number loss.

#### Pathway analyses

**Figure 4:**
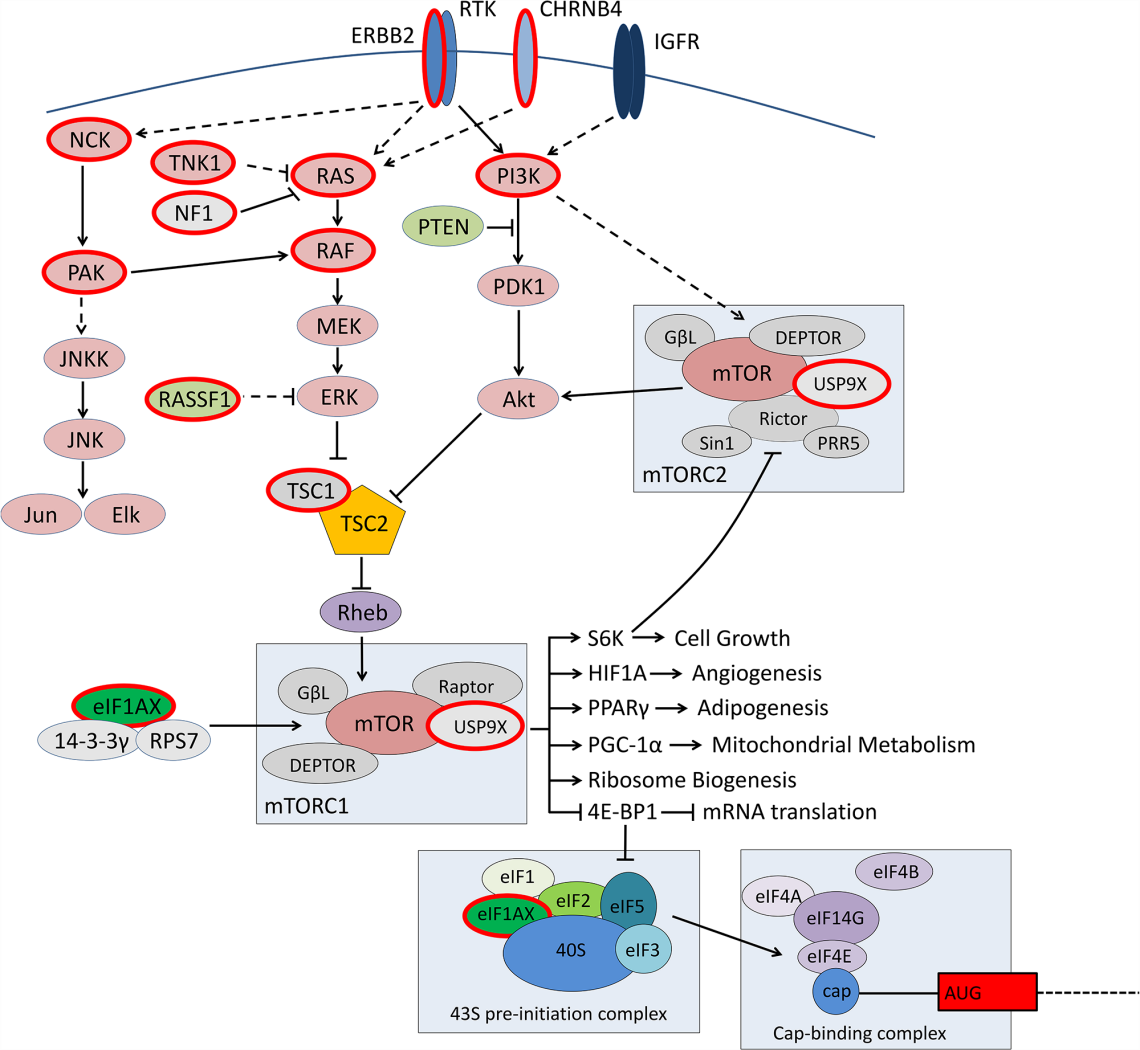
Molecular drivers of low grade serous ovarian tumours Proteins circled in red indicate mutated genes identified in this study. Along with the central components of the ERBB2/RAS/RAF/MAPK pathway, a number of tumours also carried concurrent mutations in regulators of the pathway.

Pathway analyses identified MAPK/ERBB2 signalling pathways and regulators (Figure 4), and RAS/RAF/ERBB2-dependent cancer-associated pathways as significantly enriched. When these genes were removed from the analysis, a variety of regulation of gene expression mechanisms were found to be significantly enriched based on mutations in the methyltransferases and demethylases *DOT1L, KMT2A, KDM5C, KDM5A*, and the SMRT complex component *NCOR2* and the SWI/SNF components *SMARCA4*. Other genes not identified in this pathway analysis but with established roles in transcription regulation, RNA pol II regulation or mRNA regulation included *HCFC1, MED12L, ANKRD11, SMYD3* and *DHX34*.

## DISCUSSION

### *BRAF, KRAS, NRAS* and *ERBB2* oncogenic mutations are not biologically equivalent

In this study, the total number of samples with a mutation in *KRAS, BRAF, HRAS* or *ERBB2* was significantly lower in LGSCs (7/19) than SBTs (47/57, *p* < 0.001, FET). In contrast, *NRAS* mutations were not observed in the SBT cohort, possibly indicating a much greater oncogenic potential. *NRAS* mutations were detected in 26% of LGSCs, in keeping with previous reports [6]. Our findings support earlier observations that *BRAF* mutations are less frequent in LGSC and are associated with earlier stage, better outcomes and a lower likelihood of recurrence in SBTs and LGSCs [7, 20, 21]. *ERBB2* mutations have not been reported in LGSC, which may indicate a lower oncogenic potential, but may simply reflect under-investigation.

Bilaterality in SBTs has been reported in approximately one-third of cases [22] and molecular evidence supports a common clonal origin for the cells of the majority of these bilateral tumours [9, 23]. Bilaterality is likely to be a direct result of tumour spread, corresponding with stage, therefore bilateral SBT or LGSC lesions should rarely be considered as independent lesions. The *RAS/RAF* mutation status of SBTs was found to correlate with tumour stage and specific genomic aberrations, suggestive of molecular subtypes within this group. The significant and differential association of *KRAS* mutations with bilaterality/tumour spread and *BRAF/ERBB2* mutations with genomic stability suggests that despite all of these gene products being associated with the RAS/MEK/ERK pathway, mutant forms of these genes are not biologically equivalent in this context. The tendency of *KRAS* mutants to have genomic CNAs and the *BRAF* mutants to have none or very few may be explained in part by their respective relationships with RAF-1 (CRAF), since increased activity of RAF-1 is associated with aneuploidy induction. Oncogenic KRAS and NRAS have each been demonstrated to induce RAF isoform switching to activate RAF-1, but not BRAF [24].

In addition to our LGSC cohort, we also report here are a small number of serous carcinoma cases with coinciding *RAS* and *TP53* mutations, all of which were high grade-like in their genomic characteristics (Supplementary Figures S3 and S4). It is unclear whether these high grade-like tumours followed the typical HGSC progression model with early *TP53* mutations and represent the rare instances that randomly acquire *RAS* mutations, or whether they progressed from SBTs and LGSCs, acquiring late *TP53* mutations and subsequently HGSC-like genomic complexity. The mixed grade carcinoma IC499, predominantly grade 1 with foci of grade 2/3, may represent a LGSC that has progressed to high grade as it was found to carry an *EIF1AX* mutation (along with *NRAS* and *TP53* mutations), which have only been reported in the 316 ovarian carcinomas assessed by the TCGA [12] in a case later reviewed as likely LGSC (TCGA-61-2095) [13].

### Progression of SBT to LGSC may be associated with distinct molecular events

The global level of CNA was higher in LGSCs than SBTs, however a subset of the SBTs in this study had equivalent levels of aberrations to LGSCs, raising the possibility that this may be a predictor of tumours that are likely to progress or recur. In addition, specific genomic aberrations were identified that were unique to, or highly enriched in, the LGSCs, indicating a molecularly identifiable transition from borderline tumour to carcinoma and possible predictors for likelihood of progression. However, our samples were not matched SBT-LGSC pairs, thus at present our findings are correlative. For some of these alterations, such as loss of 1p or 22, the underlying driver gene is not known. Exome sequencing did not identify any homozygously mutated genes in these regions, so genes affected by haploinsufficiency or promoter methylation may be the targets. In contrast, 9p LOH has a clear candidate gene in *CDKN2A*, particularly given the highly focused homozygous deletions observed.

Only one of the 57 SBTs was observed to have an LOH event targeting *CDKN2A* compared to 53% of LGSCs. The increased rate of loss of 9p/9p21.3 in LGSCs compared to SBTs suggests that loss of p16/ARF/p15 activity may be integral to progression from borderline tumour to carcinoma. Immunohistochemistry for p16 does not entirely reflect the relationship observed at the copy number level for LGSC. Although a trend was observed for decreasing p16 staining from SBT to LGSC, generally the patterns observed in low grade tumours are highly heterogeneous (Supplementary Figures S1 and S2). This staining heterogeneity may reflect some level of tumour (epi) genetic heterogeneity or potentially indicates that it is the combinatorial loss of p16/ARF/p15 that is important. Our results are consistent with previous reports of p16 staining as a differential between LGSC and HGSC [25, 26]. It is clear from these results, however, that p16 IHC cannot be used to differentiate SBTs from LGSCs, consistent with the findings of Altman *et al*. (2013).

### Novel molecular drivers

Although LGSCs have long been known to be characterised by *KRAS* and *BRAF* mutations, 20–30% of SBTs and 55–75% of LGSCs are wildtype for these archetypical mutations. In this and a previous study we identified *NRAS* mutations in an additional ~20% of LGSCs but still leaving a sizable proportion of cases with no known mutation. It remains to be determined whether other RAS/MAPK pathway members are mutated in these “wildtype” tumours. Three tumours contained mutations in ERBB2/RAS/RAF/MEK pathway members or regulators: an *ARAF* mutation in combination with a *NCK1* mutation, a *PAK1* mutation in combination with a *NF1* mutation, and an *ERBB2* mutation in combination with a *TSC1* mutation. However, some of these and other potential alternative driver genes identified in wildtype tumours such as *KMT2A, FANCC* and *ERC1* were also identified in many of the RAS/RAF mutant cases (Figure 3). The drivers of these wildtype tumours therefore remain unclear. It is possible that mutations in genes outside of the exome targeted here are responsible, or that larger-scale CNA, inversions or translocations may be important, especially given the trend towards wildtype tumours harbouring more extensive CNAs. These possibilities would be best tested by whole-genome sequencing of a larger cohort, however, we did note that *KRAS*/*BRAF*/*HRAS*/*ERBB2*/*NRAS*-wildtype tumours were enriched for loss of heterozygosity on 17q in both the SBT and LGSC cohorts (combined *p* = 0.005 FET), which is potentially associated with an alternative driver such as NF1 although only a single truncating *NF1* mutation was detected in this study.

Exome sequencing identified two novel candidate genes, *EIF1AX* and *USP9X*, which are enriched in LGSC compared to SBT, suggesting they may be key contributors to carcinogenesis, although the biological impacts of mutant *EIF1AX* and *USP9X* in ovarian tumours remains to be determined. Mutations in these genes were almost exclusively identified in *BRAF/KRAS/NRAS* mutant tumours, suggestive of cooperative biological effects.

*EIF1AX* has recently been reported as recurrently mutated in uveal melanomas, in mutual exclusion with mutations in *SF3B1* [27]. Through exome and targeted sequencing we identified somatic *EIF1AX* mutations in 1.7% of SBTs and 15% of LGSCs, and one mixed grade carcinoma. These mutations were mutually exclusive with a single *SF3B1* p.K700E hotspot mutation identified in a LGSC. *EIF1AX* mutations have been detected at low frequency in a variety of cancer types reported in the TCGA studies (1.6% endometrial carcinoma, 1.5% thyroid carcinoma, 1.4% low grade glioma, 1.3% lung adenocarcinoma, 1.1% cutaneous melanoma (cBioPortal) [28]. EIF1AX forms a core component of the eukaryotic translation initiation complex [29] and a potential promoter of mTOR activity as part of a complex with 14-3-3γ and RPS7 [30] (Figure 4). The functional effects of *EIF1AX* mutation are unknown, but are anticipated to have a global impact on protein production. Interestingly, a rare *EIF1AX* 5′ UTR germline SNP (rs201653081) was identified in one SBT case, the same SNP being recently identified in anaplastic thyroid carcinomas, which show some overlap in their mutation spectrum with LGSC. The 5′ UTR SNP is predicted by MutationTaster [31] to be disease causing raising the possibility that germline mutations in *EIF1AX* and other candidate genes could potentially genetically predispose to this tumour type, which may explain the typically younger age of diagnosis compared to other subtypes of ovarian cancer.

*USP9X* (ubiquitin-specific peptidase 9X) mutations were identified in 2.6% SBTs and 11% LGSCs, and a focal deletion in a mixed-grade carcinoma. USP9X has been found to regulate the stability of a diversity of proteins, and potentially as a result of this appears quite context-dependent in its function, sometimes with oncogenic characteristics and sometimes tumour suppressive. In prostate cancer cells USP9X has been found to deubiquitinate and stabilise ERG [32], while increasing expression of USP9X was found to correlate with higher grade and poorer outcome in oesophageal squamous cell carcinomas [33]. In contrast, *USP9X* was found to be targeted by recurrent truncating mutations and deletion in gingivo-buccal oral squamous cell carcinoma [34] characteristic of a tumour suppressor gene, and reduced expression has been associated with poorer survival and increased metastatic burden in pancreatic ductal adenocarcinoma patients [35]. *USP9X* mutations have been identified in several cancer types at significant levels in the TCGA studies: 11% of endometrial carcinoma, 9% of stomach adenocarcinoma and 9% of head and neck squamous cell carcinomas (cBioportal) [28]. USP9X has been isolated as a component of both mTORC1 and mTORC2 complexes, and potentially functions as a negative regulator of mTOR [36] (Figure 4), with links to anoikis resistance and chemotherapy response in multiple cancer types [35, 37, 38] warranting further investigation to assess the likely impact on new treatments being trialled in LGSC.

## MATERIALS AND METHODS

### Tissue samples and DNA extraction

Clinical features of the cohort of 57 histologically confirmed SBT and 19 LGSC (of which two cases were recurrences of prior SBTs) are summarised in Table 1. Fresh-frozen tissue samples were used for copy number and mutation analyses. All samples were collected with the patient's informed consent and the study was approved by the Human Research Ethics Committee at the Peter MacCallum Cancer Centre. Patients with ovarian tumours were identified through four primary sources: a) hospitals in the Wessex Region, UK (*n* = 7 SBT, *n* = 41 serous carcinoma) [39], b) the Australian Ovarian Cancer Study (AOCS) (*n* = 50 SBT) [5], c) Prince Henry's Institute, Victoria, Australia (*n* = 4 serous carcinoma) and the OvCaRe Tissue Bank, Vancouver, Canada (*n* = 12 serous carcinoma). The AOCS (http://www.aocstudy.org) was approved by the Human Research Ethics Committees at the Peter MacCallum Cancer Centre, Queensland Institute of Medical Research, University of Melbourne and all participating hospitals.

Each case was independently reviewed by two pathologists (RS, PM, CBG, PA). Pathology review was conducted on cryosections adjacent to the tissue from which DNA was extracted and where possible from multiple other diagnostic formalin-fixed, paraffin-embedded tumour blocks. Microdissection and DNA extraction were performed as previously described [40], briefly, epithelial and adjacent stromal tissue were needle microdissected from H&E stained sections (Supplementary Figure S7, Supplementary Tables S1 and S2).

### Copy number data and analysis

SBTs and LGSCs were analysed using Affymetrix SNP6.0 Human Mapping arrays according to the protocol recommended by the manufacturer, with the exception that the input was reduced from 500 ng to 250 ng and the reaction volumes were reduced by 50% for all processes prior to the SNP6.0 PCR step. Reduction in DNA input does not result in any loss in data quality [41]. All previously unpublished SNP data has been made publicly available through Gene Expression Omnibus (GSE58579, http://www.ncbi.nlm.nih.gov/geo/). Copy number analysis was performed as previously described [40], using Nexus Copy Number™ 7.0 Discovery Edition (BioDiscovery, Inc.) and Partek^®^ Genomics Suite 6.5.

### Mutation screening

Mutation screening was performed by Sanger sequencing using whole-genome amplified DNA as previously described [40]. All samples were assessed at *BRAF* exons 11 and 15, *KRAS* codons 12, 13 and 61, *HRAS* codons 12, 13 and 61, *ERBB2* exon 20, *TP53* exons 4–9, and *CDKN2A* exons 1 and 2 (LGSCs only). *NRAS* mutation data for the 47 AOCS SBTs was derived from Emmanuel *et al*. [6], the remaining 10 SBTs and the LGSCs were all sequenced for *NRAS* codons 12, 13 and 61. Sanger screening was also performed for the following candidate genes: *SF3B1* (NM_012433.2) exons 14, 15 and 16; *EIF1AX* (NM_001412.3) exons 1 and 2; *USP9X* (NM_001039590.2) exons 2–45; *RNASE1* (NM_002933.4) exon 2; *NCK1* (NM_006153.4) exons 2–4. Primers were designed using Primer3 [42] and are listed in Supplementary Table S8. All mutations were validated in non-whole-genome amplified DNA.

### Microsatellite instability

Microsatellite stability testing was performed using the NCI-recommended reference panel of five microsatellite markers (BAT25, BAT26, D2S123, D5S346, D17S250) and an additional four microsatellite markers (see Supplementary Table S8 for oligonucleotide details).

### Exome sequencing and bioinformatics

For each case 500 ng − 1 μg of microdissected tumour DNA and matched lymphocyte DNA (in one case stromal DNA was used) was sheared to < 1000 bp using a Covaris^®^ ultra-sonicator (Covaris^®^), libraries prepared using the Illumina TruSeq DNA Sample Preparation procedure (Illumina), and enriched for exome sequencing using the SeqCap EZ Human Exome Library v2.0 (Roche NimbleGen). Exomes were sequenced with 100 bp PE reads in pools of three per lane on a HiSeq2000 (Illumina), to a 140x mean coverage of target bases, with > 90% of target bases covered at 10x for all samples (average 95.6%), and an average of 90% of target bases with 1/5 of the mean coverage.

Sequence reads were aligned to the human genome (GRCh37/hg19) using BWA-MEM (v0.7.7-r441) [43]; duplicates marked using Picard (v1.77); local indel realignment and base quality recalibration performed using GATK (v2.7-2-g6bda569) [44]; indel detection performed using GATK Unified Genotyper (v2.7-2-g6bda569), Indel Genotyper, Pindel (v0.2.5a3) [45], and VarScan2 (v2.2.4) [46]; SNV prediction performed using GATK Unified Genotyper, MuTect (v2.7-1-g42d771f) [47], SomaticSniper [48], JointSNVMix2 (v0.8-b2) [49], and VarScan2 (v2.2.4); and variants annotated using Ensembl variant effect predictor v73.

Variants were enriched for somatic events by filtering for > = 0.05 alternate allele frequency in the tumour and < 0.05 alternate allele frequency in the germline. Variants were additionally filtered for those with >= 3 reads supporting the alternate allele and called by >= 3 variant prediction algorithms, and filtered against a database of 250 in-house exomes to remove common variants and artefacts. This more stringently filtered list of somatically mutated genes was subsequently used to identify potentially recurrently mutated genes where additional variants had not reached the stringent thresholds.

Pathway and functional mutation enrichment analyses were performed using IntOGen Mutation Analysis v2.4.1 [50] and MetaCore™ v6.0 Enrichment Analysis Workflow for genomic variants (Thomas Reuters).

### Statistical analyses

Associations between mutations and CNAs or histological features were determined using Fisher's Exact Test (FET). For the purposes of this study tumour stage was considered as stage I or > stage I (stage II–IV). Mann-Whitney tests were used to assess the difference in mean fraction of the genome altered (FGA) and intrachromosomal breakpoints between tumour groups using GraphPad Prism version 6.01 (GraphPad Software, La Jolla California USA).

### RNA extraction and cDNA synthesis

To determine the effect of potential splice site mutations fresh-frozen tissue was needle microdissected from cresyl violet acetate stained sections. RNA was extracted using the Qiagen RNeasy kit (Qiagen) as per the recommended protocol. First-strand cDNA synthesis was performed with 0.1–1 ug total RNA using SuperScript III Reverse Transcriptase (Life Technologies), with both oligo(dT)_18_ and random hexamers (Bioline). Primers spanning the intron-exon junctions were designed using Primer3 [42] and are listed in Supplementary Table S8.

## CONCLUSIONS

We have identified a correlation between the specific oncogenic activation and tumour spread beyond the ovary in SBT. We also identified a number of genomic CNAs and mutations in novel genes that are enriched in or unique to LGSCs compared to SBTs, indicating that there are key identifiable molecular events underlying this transition, including novel mutations in *EIF1AX* and *USP9X*. These findings suggest the possibility of using patient-specific molecular events to improve treatment practices to best suit the clinical behaviour of the tumour, although larger studies with clinical follow-up are required. Long term clinical management is an important consideration as although patients have a very good short-term prognosis, over an extended period recurrence and progression of SBTs to LGSCs can be significant and later stage LGSCs are often chemoresistant.

## SUPPLEMENTARY MATERIALS AND METHODS








