# Supplementary material for: Molecular profiling of low grade serous ovarian tumours identifies novel candidate driver genes
Source: Oncotarget. 2015 Oct 19;6(35):37663–77. doi: 10.18632/oncotarget.5438 (PMC4741956; doi:10.18632/oncotarget.5438)
Supplement: Supplementary file 1 [file oncotarget-06-37663-s001.pdf]

SUPPLEMENTARY MATERIALS AND METHODS

Microdissection and DNA extraction

Matched normal DNA extracted from peripheral lymphocytes was used for comparison with all SBT cases except IC510, 8591 and 1408, where adjacent normal tissue was dissected from the tumour block. For the serous carcinomas, only 7/21 cases had matched normal DNA from lymphocyte available and sufficient quantities of normal tissue were not available.

Mutation screening

Mutation information for *KRAS* exons 12 and 13, *BRAF* exon 15, *ERBB2* exon 20 and *TP53* exons 5–8 have been previously published for 24 of the SBTs[18]. For case PHI33–07 *TP53* sequencing was extended to exons 2–11.

Copy number data

Copy number analysis has previously been published for 24 of the SBTs[18], indicated in supplementary Table S1. Additional SNP6 data was obtained for 14 cases of LGSC and seven cases of high grade (grade 2–3) serous carcinoma (HGSC). Previously published raw SNP6 copy number data was obtained for four LGSCs and 33 HGSCs from Gorringe *et al*. (2010)[36] and for 316 HGSCs from The Cancer Genome Atlas (TCGA, 2011)[37].

Copy number analysis

All samples were analysed using unpaired normalisation against a pool of reference samples consisting of all blood normals from the cohort. Samples with paired blood normals or stromal normals with no CNAs were also analysed using paired normalisation. Fraction of the genome altered (FGA) and intrachromosomal breakpoint counts were performed using the circular binary segmentation output (minimum of 10 probes) on total copy number in Partek. For these metrics regions < 1 Mb were removed to reduce the influence of germline copy number polymorphisms and noise.

p16 immunohistochemistry and scoring

Three micron sections of tissue microarray (TMA) (Gorringe cases[36] and AOCS[11,17]) or frozen tumour rapidly fixed in acetone were stained using antibodies for p16 (clone E6H4, CINtec®, Ref 9511, Ventana/Roche, Tucson AZ). Scoring was performed using a ternary system: 0 = negative (< 10% positively stained cells), 1 = normal staining (strong patchy or weak diffuse), 2 = strong block staining (> 90% cells positive). For example staining see Supplementary Figures S1 and S2.

p16 immunohistochemistry

The *CDKN2A* locus appears to be the primary target of the highly enriched copy number imbalance and loss events targeting the short arm of chromosome 9, accompanied by focal homozygous deletion events and truncating mutations. Immunohistochemical (IHC) staining for $p16^{INK4A}$ was performed for 30 SBT, 16 LGSC and 191 HGSC on TMAs, and 9 LGSC with whole sections; 26 of these cases have overlapping copy number data (Supplementary Table S1). $p16^{INK4A}$ staining was typically found to be highly heterogeneous within each individual tumour of the SBT and LGSC cohorts, with ~40–50% of cases having strong patchy or weak diffuse staining (Supplementary Figures S1 and S2; Supplementary Tables S4 and S5). There appeared to be a trend towards negative staining and homogeneous low level staining in the LGSCs compared to SBTs, and carcinomas with 9p loss had negative staining (Supplementary Table S5). In contrast 80% of HGSCs were scored with 100% positive or 100% negative staining (Supplementary Table S4).

SUPPLEMENTARY FIGURES AND TABLES

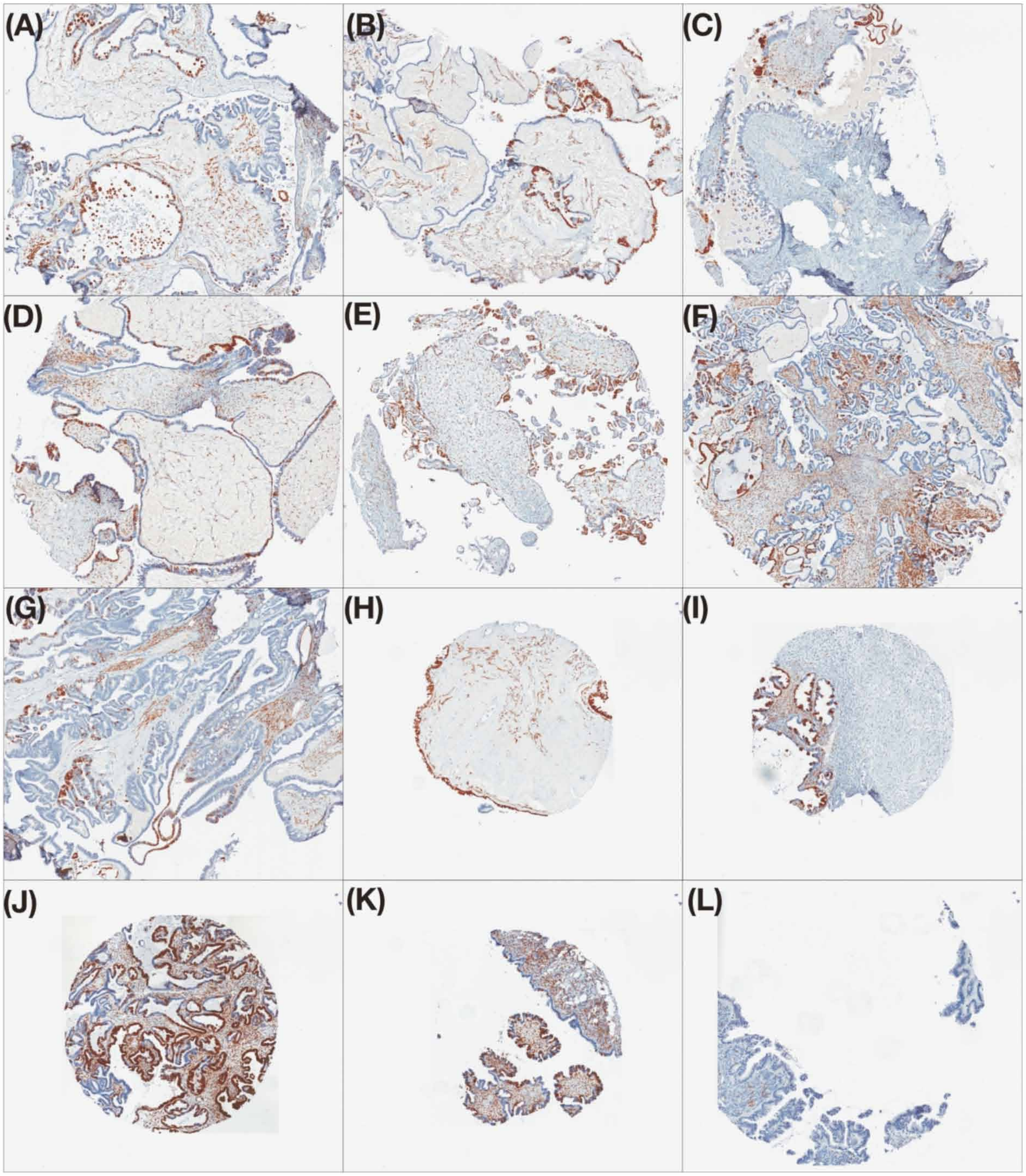

Supplementary Figure S1: Serous borderline tumours p16 IHC. A. 2006 [1], **B.** 2007 [1], **C.** 2952 [1], **D.** 1277 [1], **E.** 2408 [1], **F.** 4243 [1], **G.** 4808 [1], **H.** 1025 [2], **I.** 1457 [1], **J.** 4267 [1], **K.** 6543 [1], **L.** 9876 [0]. 5x magnification. [Indicates ternary staining score].

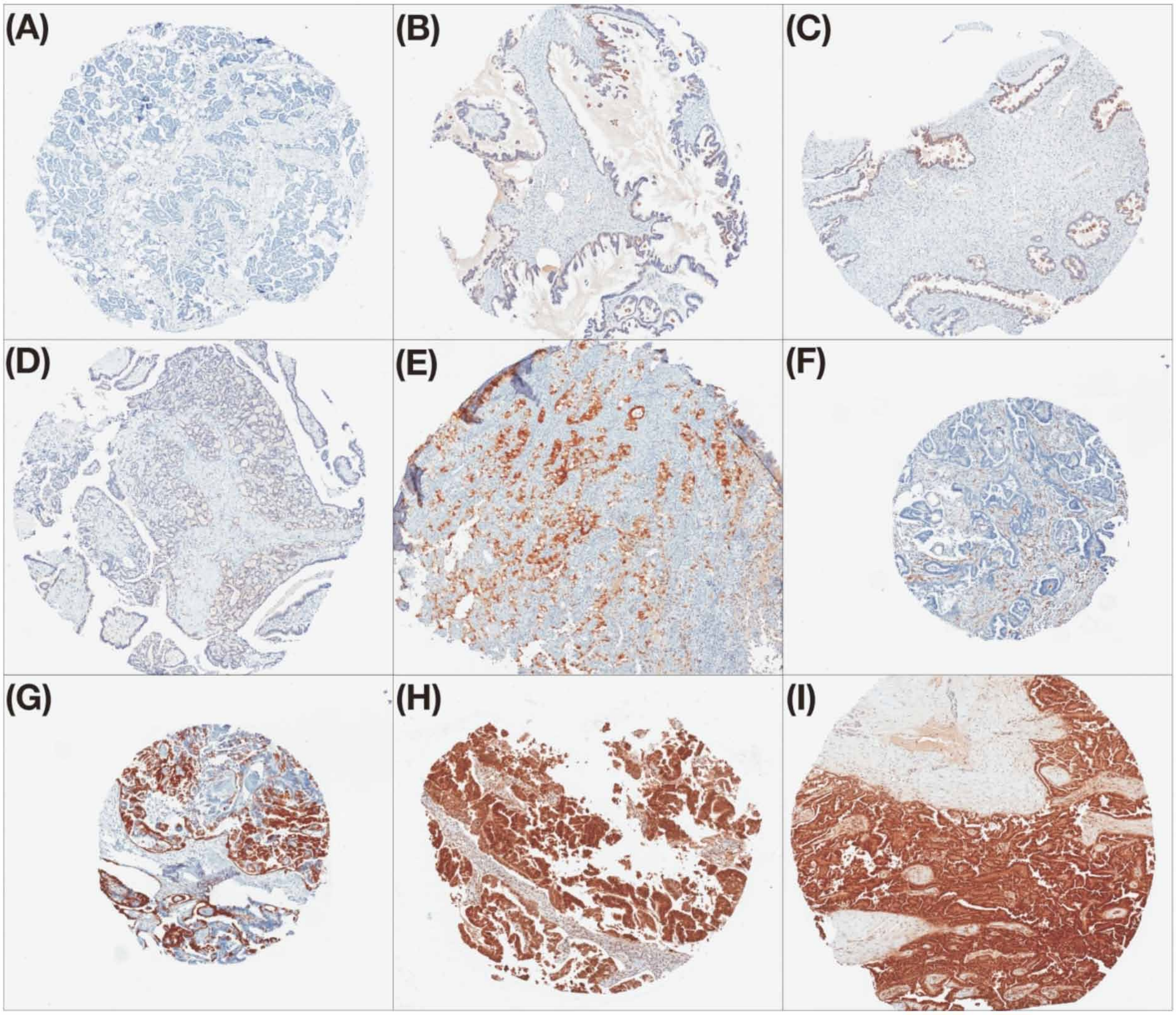

Supplementary Figure S2: Serous carcinoma p16 IHC. LGSC – **A.** IC471 [0], **B.** IC462 [1], **C.** IC487 [1], **D.** IC548 [1], **E.** 3288 [1], **F.** 2021 [0], **G.** 6430 [1]; HGSC - **H.** IC499 [2], **I.** IC565 [2]. 5x magnification. [Indicates ternary staining score].

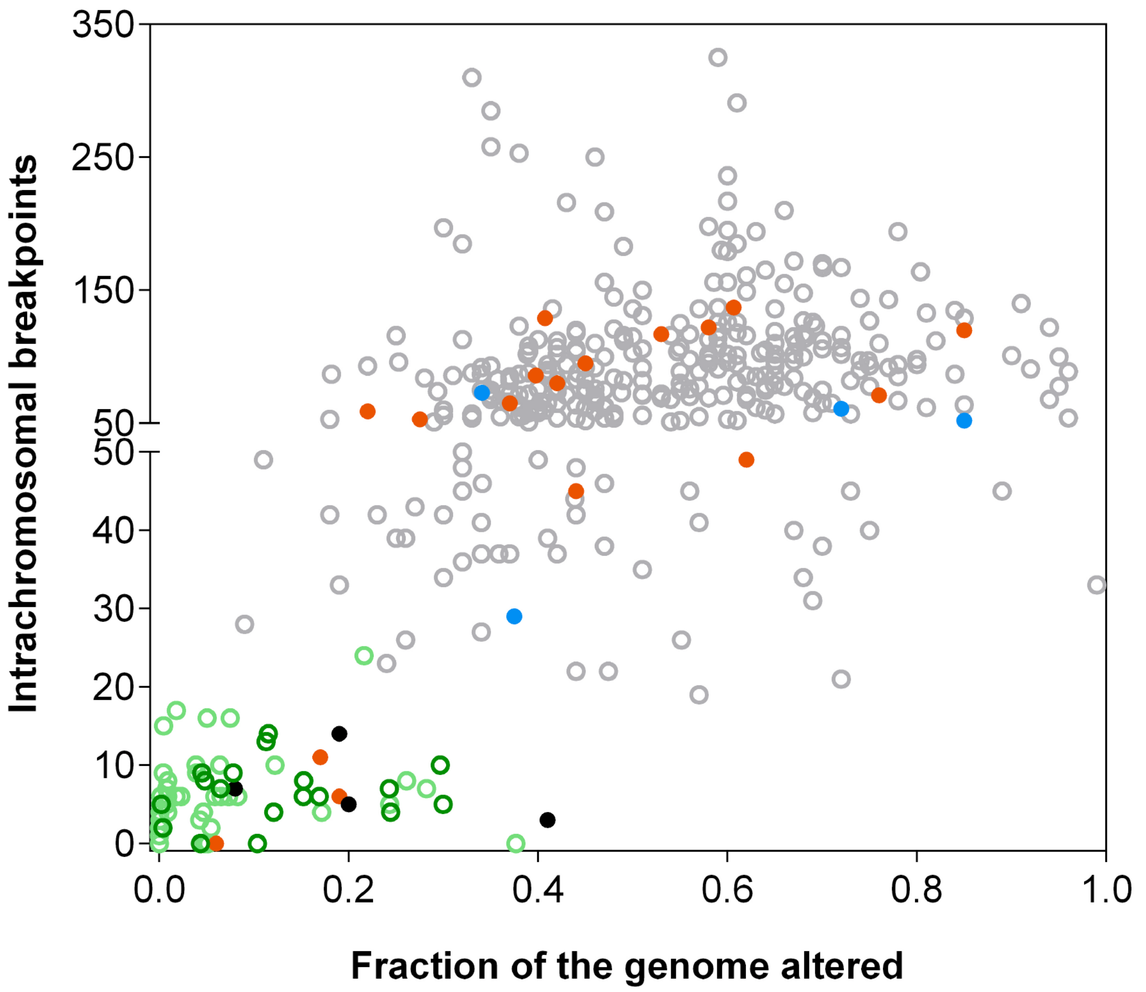

Supplementary Figure S3: Combined genomic aberration level. SBTs (open light green) and LGSCs (open dark green) cluster together. HGSCs with no identified *TP53* mutation (closed black), *RAS/RAF* mutants with co-occuring *TP53* mutation (closed blue), HGSCs with *TP53* mutations (open grey) and TCGA cases with *RAS/RAF* mutations and wild-type *TP53* (closed orange). The four *RAS/BRAF* mutant HGSCs with a *TP53* mutation (closed blue) cluster more closely with the combined HGSC cohort (open grey). NB: Y-axis is split at 50 breakpoints and differs in scaling. All SBT and LGSC cluster together, with the exception of a single SBT that harbors a *KRAS*^{G12V} mutation concurrent with high-level amplification across 12p12-p13.

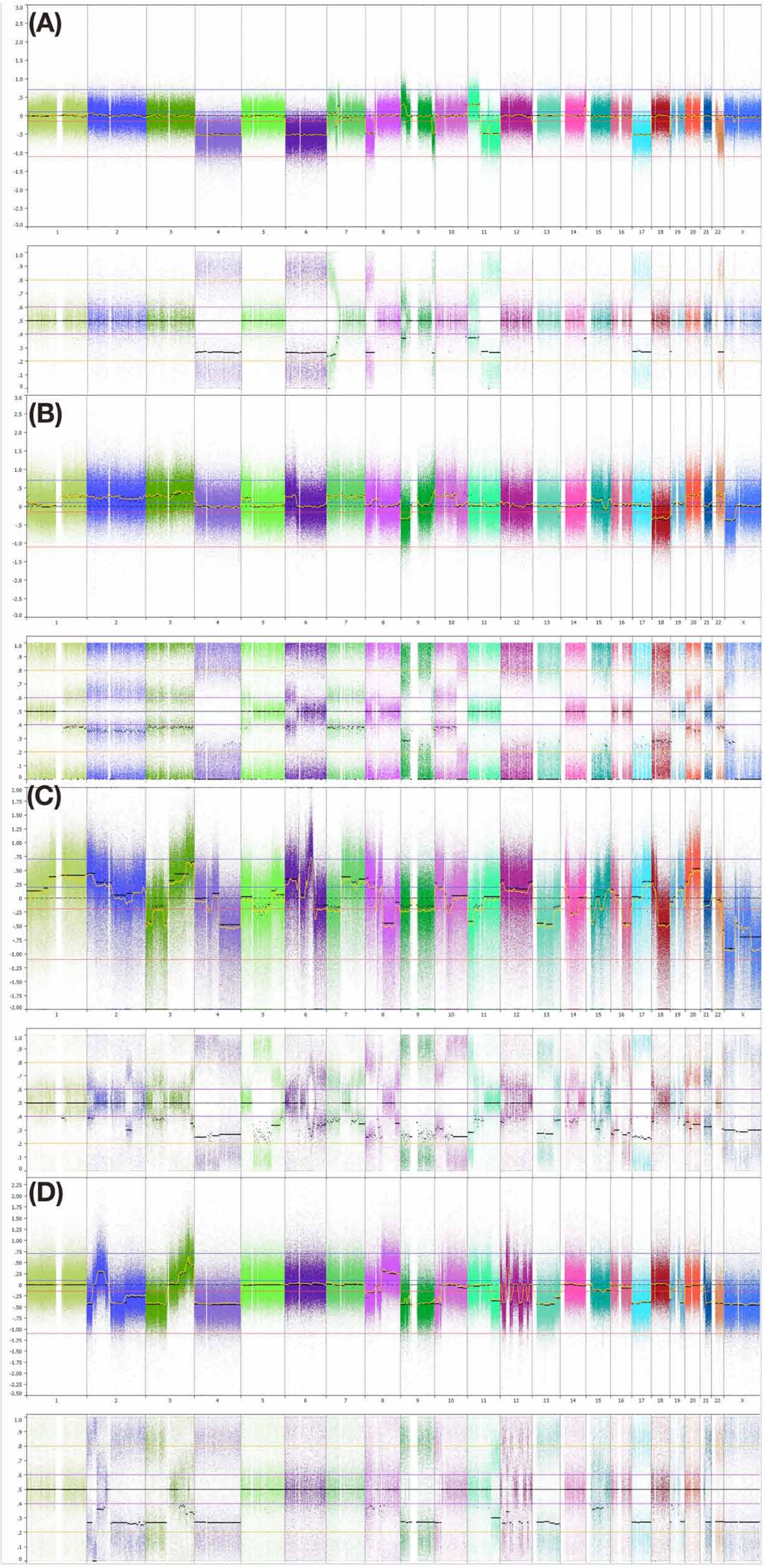

Supplementary Figure S4: *TP53-RAS* mutant tumours copy number plots. A. Case IC499, *NRAS* p.(Q61R), *TP53* p.(W146*). **B.** Case VOA1841, *KRAS* p.(G12V), *TP53* p.(R175H). **C.** TCGA-04–1347 *TP53* p.(S215R), *BRAF* p.(S637*) **D.** TCGA-61–2016 *TP53* p.(E204*), *NRAS* p.(Q61R) Upper plots indicate total copy number; lower plots indicate B-allele frequency.

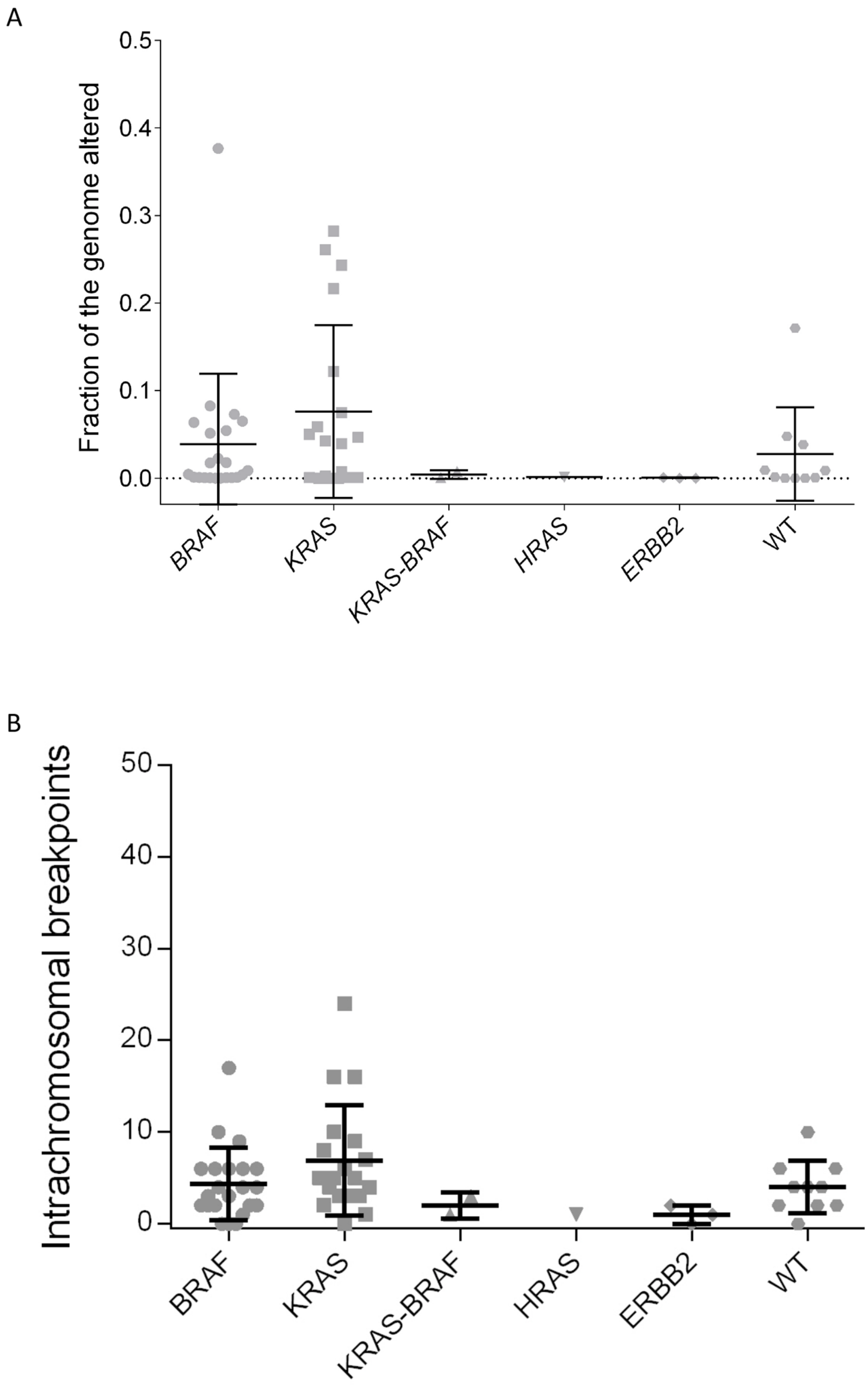

Supplementary Figure S5: Fraction of the genome altered A. and Intrachromosomal breakpoints B. by oncogenic mutation in SBT. Mean and standard deviation of the mean plotted by oncogenic mutation.

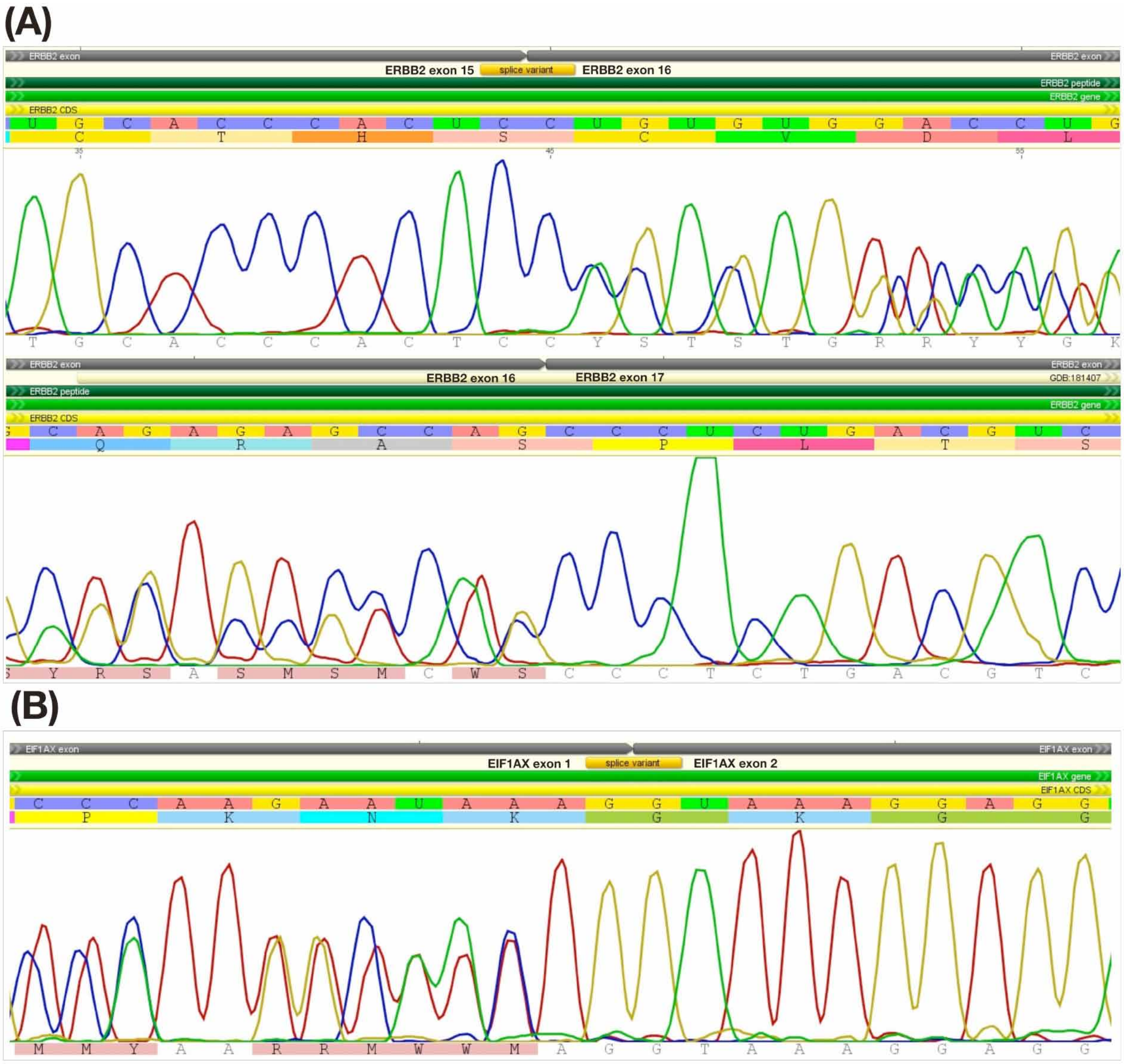

Supplementary Figure S6: cDNA sequencing of *EIF1AX* and *ERBB2* splice-region mutationS. A. The VOA133T *ERBB2* c.1899–1G > A ESS mutation was predicted by HSF v2.4.1 to reduce the exon length by 1bp, however, cDNA sequencing determined that this mutation results in skipping of exon 16 (NM_004448), which encodes part of the growth factor receptor IV domain. **B.** The *EIF1AX* (NM_001412.3) mutation in tumour 7198T c.17–7G > C was predicted and validated to extend exon 2 by 6bp, maintaining the reading frame and introducing an Alanine and a Serine residue (p.(K5_G5insAS)).

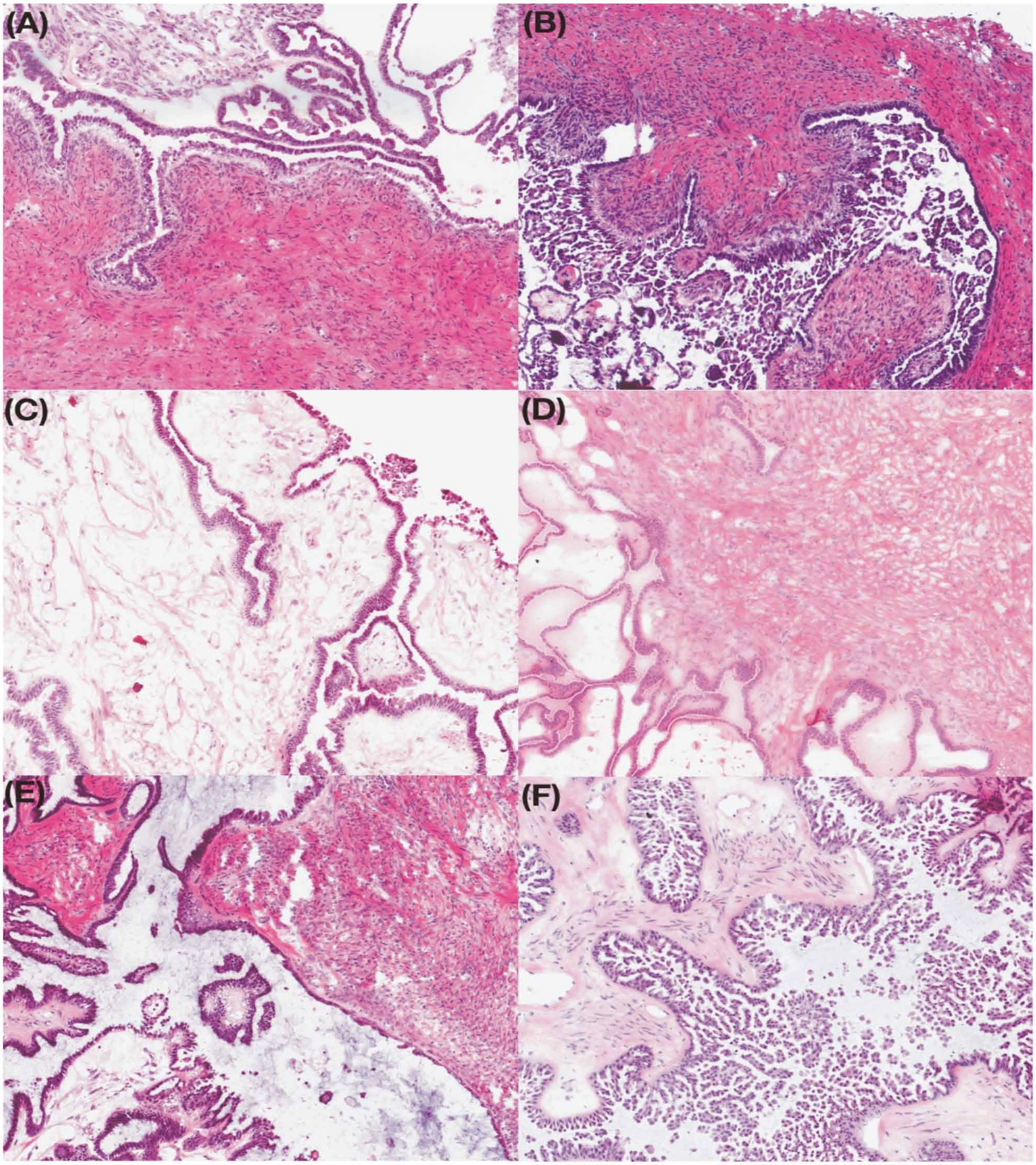

Supplementary Figure S7: Representative haematoxylin and eosin stained sections. **A–B.** Wildtype tumours (2615 and 2088) with stromal copy number aberrations. **C.** *KRAS* mutant tumour (11241). **D.** *BRAF* mutant tumour (IC486). **E–F.** *KRAS-BRAF* double mutant tumours (2072, 3905). 10X magnification.

Supplementary Table S1: Serous borderline tumour mutation and copy number data.

Supplementary Table S2: Low grade serous carcinoma mutation and copy number data.

Supplementary Table S3: Recurrent copy number aberrations in LGSCs compared to SBTs

	SBT (*n* = 57)	LGSC (*n* = 19)	Fisher's exact test *P*-value
Gains			
1q	4 (7%)	3 (16%)	0.3571
3	1 (2%)	3 (16%)	0.0461
7/7q	10 (18%)	4 (21%)	0.7399
8/8q	10 (18%)	4 (21%)	0.7399
12/12p	9 (16%)	4 (21%)	0.7260
13/13q	0	3 (16%)	0.0138
Losses			
**1p**	**6 (11%)**	**9 (42%)**	**0.0013**
**9/9p**	**2 (3.5%)**	**10 (53%)**	**< 0.0001**
**9q**	**1 (2%)**	**5 (26%)**	**0.0032**
17p	1 (2%)	3 (16%)	0.0461
17q	3 (5%)	4 (21%)	0.0611
**18q**	**1 (2%)**	**5 (26%)**	**0.0032**
19	5 (9%)	4 (21%)	0.2155
**22**	**0**	**6 (32%)**	**0.0001**
X	1 (2%)	3 (16%)	0.0461

Bold: Bonferroni adjusted α = 0.0033. Regions of CNA in > 15% of LGSC included for comparison.

Supplementary Table S4: p16^{INK4A} IHC scoring

p16 staining pattern	SBT (*n* = 30)	LGSC (*n* = 25)	HGSC (*n* = 192)
0	3 (10%)	6 (24%)	28 (15%)
1	23 (77%)	19 (76%)	37 (19%)
2	4 (13%)	0	128 (66%)

Scoring: 0 = negative (no or < 10% positively stained cells), 1 = normal staining (strong patchy or weak diffuse), 2 = strong block staining (> 90% cells positive).

Supplementary Table S5: LGSC p16 immunohistochemistry

Sample ID	Histology	*RAS/RAF* mutation	*CDKN2A* CN state	p16 IHC [score]	Tissue
1268	SBT	$BRAF^{V600E}$	Neutral	Strong diffuse[2]	TMA
2006	SBT	$BRAF^{V600E}$	Neutral	< 10% positive [0]	TMA
2007	SBT	$BRAF^{V600E}$	Neutral	Patchy [1]	TMA
2713	SBT	$KRAS^{G12D}$	Neutral	Strong diffuse [2]	TMA
2952	SBT	$KRAS^{Q61H}$	Neutral	< 10% positive [0]	TMA
1277	SBT	$ERBB2^{M774\_A775insAYVM}$	Neutral	Patchy [1]	TMA
4808	SBT	$ERBB2^{M774\_A775insAYVM}$	Neutral	Patchy [1]	TMA
7315	SBT	$BRAF^{V600E}$	Neutral	Patchy [1]	TMA
4452	SBT	$KRAS^{G12D}$	Neutral	Patchy [1]	TMA
1457	SBT	$BRAF^{V600E}$	Neutral	Patchy [1]	TMA
9663	SBT	$BRAF^{V600E}$	Neutral	Negative [0]	TMA
IC462	LGSC	$KRAS^{G12D}$	Neutral	Weak diffuse [1]	TMA
IC471	LGSC	WT	HD	Negative [0]	TMA
IC482	LGSC	$KRAS^{G12R,G12V}$	Neutral	< 10% positive [0]	TMA
IC487	LGSC	$BRAF^{V600E}$	Neutral	Weak diffuse [1]	TMA
IC548	LGSC	WT	Neutral	Weak diffuse [1]	TMA
IC586	LGSC	$BRAF^{V600E}$	Neutral	Negative [0]	Acetone fixed frozen
IC325	LGSC	$NRAS^{Q61R}$	Neutral	Not tested	
P4085	LGSC	$NRAS^{Q61R}$	Neutral	Not tested	
PHI679–07	LGSC	$NRAS^{Q61R}$	Neutral	< 10% positive [0]	Acetone fixed frozen
VOA221	LGSC	WT	Neutral	Negative [0]	Acetone fixed frozen
VOA324	LGSC	WT	LOH	< 10% positive [0]	Acetone fixed frozen
VOA617	LGSC	$BRAF^{V600E}$	LOH	< 10% positive [0]	Acetone fixed frozen
VOA814	LGSC	$KRAS^{G12R}$	LOH	Patchy [1]	Acetone fixed frozen
VOA875	LGSC	WT	CNLOH	Negative [0]	Acetone fixed frozen
VOA1056	LGSC	$NRAS^{Q61R}$	HD	Not tested	
VOA1133	LGSC	WT	LOH	Not tested	
VOA1177	LGSC	$KRAS^{G12R}$	HD	Not tested	
VOA1802	LGSC	WT	LOH	< 10% positive [0]	Acetone fixed frozen
PHI33–07	LGSC	WT	LOH	< 10% positive [0]	Acetone fixed frozen
IC499	Mixed Grade SC	$NRAS^{Q61R}$, $TP53^{W146*}$	Neutral	Strong diffuse [2]	TMA/Acetone fixed frozen

WT, wildtype; HD, homozygous deletion; LOH, loss of heterozygosity; CNLOH, copy neutral LOH.

Supplementary Table S6: SBT mutation spectrum with clinical features and copy number aberrations

	KRAS (*n* = 19[1])	*BRAF* (*n* = 22[1])	*KRAS+BRAF* (*n* = 2)	*HRAS* (*n* = 1)	*ERBB2* (*n* = 3)	WT (*n* = 10)
**CNAs** (35/57 cases)	**16** ($p = 0.0199$)	11	0	0	0	8
**No CNAs** (22/57 cases)	3	11	2	1	3 ($p = 0.0526$)	2
**Stromal CNAs** (4/39 cases)	0	1	0	NT	0	**3** ($p = 0.0220$)
**Unilaterality** (35/57 cases)	**7**	17	1	1	2	7
**Bilaterality** (22/57 cases)	**12** ($p = 0.0101$)	5	1	0	1	3
**Staged** (34/57 cases)						
**Stage I**	**1**	6	0	1	1	5
**Stage II–IV**	**9** ($p = 0.0240$)	7	1	0	2	1
**1p LOH** (6/57 cases)	**5** ($p = 0.0129$)	0	0	0	0	1
**7 gain** (10/57 cases)	4	5	0	0	0	1
**7q CN LOH** (4/57 cases)	0	**4** ($p = 0.0185$)	0	0	0	0
**8/8q gain** (9/57 cases)	**6** ($p = 0.0478$)	1	0	0	0	2
**12/12p gain** (9/57cases)	**7** **($p = 0.0043$)**	0	0	0	0	2
**12p CN LOH** (2/57 cases)	2 ($p = 0.1071$)	0	0	0	0	0
**17q CN LOH** (2/57 cases)	0	0	0	0	0	2 ($p = 0.0282$)

1. Two cases carrying both an activating KRAS G12 mutation and BRAF P-loop mutations have been excluded from these numbers and considered separately as KRAS+BRAF. 2. Only 8/10 cases were able to be tested for stromal CNAs. CNAs, copy number aberrations; WT, wildtype; NT, not tested; LOH, loss of heterozygosity; CNLOH, copy neutral loss of heterozygosity. *P*-values based on two-tailed Fisher's exact test.
Bold; Bonferroni adjusted *p*-value threshold of 0.0045

Supplementary Table S7: Exome sequencing variants.

Supplementary Table S8: Primers and PCR conditions

Gene	Exon	PCR conditions	Primer 1	Primer 2
KRAS	2	Touchdown 60–54, 45 cycles	CCTAAACTCTTCA TAATGCTTGCTC	CATGAAAAT GGTCAGA GAAACC
KRAS	3	Touchdown 65–55, 45 cycles	TTTTTGAAGTAAA AGGTGCACTG	TGGCAAATA CACAAAG AAAGC
NRAS	2	Touchdown 65–55, 45 cycles	CACTAGGGTTTTC ATTTCCATTG	AATACAGAA TATGGGTAA AGATGATCC
NRAS	3	Touchdown 65–55, 45 cycles	CCCCAGGATTCT TACAGAAAA	TGTAGAGGT TAATATCCG CAAATG
HRAS	2	Touchdown 65–55, 45 cycles	GTGGGTTTGCCC TTCAGAT	AGCTGCTGG CACCTGGAC
HRAS	3	Touchdown 65–55, 45 cycles	CAGGAGACCCT GTAGGAGGA	CCTATCCTG GCTGTGTC CTG
BRAF	11	Touchdown 60–54, 45 cycles	TCCCTCTCAGGC ATAAGGTAA	CGAACAGTG AATATTTCC TTTGAT
BRAF	15	Touchdown 60–54, 45 cycles	CCTAAACTCTTC ATAATGCTTGCTC	CCACAAAAT GGATCCAG ACA
ERBB2	20	Touchdown 63–53, 45 cycles	CCACATGCCCAG CAAGAGTCC	CTACATGGG TGCTTCCC ATTC
TP53	2	Touchdown 63–53, 45 cycles	GGGTTGGAAGT GTCTCATGC	CTTCCAATG GATCCACT CAC
TP53	3	Touchdown 65–55, 45 cycles	CCATGGGACTG ACTTTCTGC	GGCAAGGG GGACTGTA GATG
TP53	4	Hotstart 64° annealing, 40 cycles	CCTGGTCCTCTGACT GCTCTTTTCACCCA	GGCCAGGC ATTGAAGT CTCAT
TP53	5	Touchdown 60–54, 45 cycles	CAACTCTGTCT CCTTCCT	TGTCGTCTC TCCAGCC CC
TP53	6	Hotstart 63° annealing, 40 cycles	AGAGACGACA GGGCTGGTTG	CTTAACCCC TCCTCCC AGAG
TP53	7	Hotstart 63° annealing, 40 cycles	CCTCATCTTGG GCCTGTGTT	AGTGTGCAG GGTGGCA AGTG
TP53	8	Touchdown 60–54, 45 cycles	CCTTACTGCCT CTTGCTTCT	ATAACTGCA CCCTTGG TCTC
TP53	9	Hotstart 64° annealing, 40 cycles	GGAGACCAAGGGTG CAGTTATGCCTCAG	CCCAATTGC AGGTAAA ACAG
TP53	10	Touchdown 65–55, 45 cycles	CATGTTGCTTTT GTACCGTCA	CAGCTGCCT TTGACCAT GAA
TP53	11	Hotstart 65° annealing, 40 cycles	TCATCTCTCCTCC CTGCTTC	GGGTTCAAA GACCCAA AACC
CDKN2A	1	Hotstart 51° annealing, 40 cycles	AGCACCGGAGGA AGAAAGAG	AGCGCTACC TGATTCCA ATTC
CDKN2A	2A	Touchdown 65–55, 45 cycles	CTTCCTTTCCGTC ATGCCG	CTCAGCCAG GTCCACG GGCA

(Continued)

Gene	Exon	PCR conditions	Primer 1	Primer 2
CDKN2A	2B	Touchdown 65–55, 45 cycles	TTCCTGGACACG CTGGTGGTG	GGAAGCTCT CAGGGTA CAAA
CDKN2A	3	Hotstart 58° annealing, 40 cycles	TGCCACACATC TTTGACCTC	AAAACTACG AAAGCGGGGTG
BAT25	n.a.	Multiplex - 55° annealing, 35 cycles	HEX-TC GCC TCC AAG AAT GTA AGT	TCT GCA TTT TAA CTA TGG CTC
BAT26	n.a.	Multiplex - 55° annealing, 35 cycles	NED-TGACTACTT TTG ACT TCA GCC	AAC CAT TCA ACA TTT TTA ACC C
D5S346	n.a.	Multiplex - 55° annealing, 35 cycles	FAM-ACTCACTCT AG TGA TAA ATC GGG	AGCAGATAA GACAGTATTACTAGTT
D17S250 (Mfd15)	n.a.	Multiplex - 55° annealing, 35 cycles	FAM-GG AAG AAT CAA ATA GAC AAT	GCTGGCCAT ATA TAT ATT TAA ACC
D2S123	n.a.	Multiplex - 55° annealing, 35 cycles	NED-AAA CAG GAT GCC TGC CTT TA	GGA CTT TCC ACC TAT GGG AC
CAT25	n.a.	Multiplex - 55° annealing, 35 cycles	NED-CCTAGAAAC CTTTATCCCTGCTT	GAGCTTGCAGTGAGCTGAGA
NR21	n.a.	Multiplex - 55° annealing, 35 cycles	GAGGCTTGTCA AGGACATAA	HEX-AATTCGGATGCCATCCAGTT
NR22	n.a.	Multiplex - 55° annealing, 35 cycles	TAAATGTATGT CTCCCCTGG	HEX-ATTCCTACTCCGCATTCACA
NR24	n.a.	Multiplex - 55° annealing, 35 cycles	GCTGAATTTTA CCTCCTGAC	HEX-ATTGTG CCATTGCA TTCCAA
EIF1AX	1	TDOWN65–55, 45 + 2.75 mM MgCl2	CCGGAAAGAA GTCAGAGACG	GAGCTCAGA GTCGCG TGTG
EIF1AX	2	TDOWN65–55, 45 cycles	ATGAAAACACT TACCCTGACCAT	AAGCCTTAA TTTCATTTTATT TCATACTGT
NCK1	2	Anneal 62, 35 cycles	GCATGTGAACT AATACTACCT CAACC	TTATGCACAC TGCTGCC AGA
NCK1	3A	Anneal 58, 35 cycles	AAGTTCATTCT TTTGGTAATC ATTTTT	CTCCATGACG ATCACCTT TG
NCK1	3B	Anneal 62, 35 cycles	CATGGCTGAGA GAGAGGATG	TGAGCTGAAT GGGTAAAGAGC
NCK1	3C	Anneal 60, 35 cycles	AGCAGCAGTCG TCAATAACC	TGATGGTTCC AAACCTGAAG
NCK1	3D	Anneal 60, 35 cycles	GCAGGAAGATC AATGGTATGG	CCCAGAGCTC TATTTGTATTTACC
NCK1	4	Anneal 58, 35 cycles	ACTGATAGAGG GCTTTCAAAATGT	CAATTTCCAAT CAAGCACGA
RNASE1	2A	Anneal 58, 35 cycles	CTGGGGGAACT GTCTGAGTC	TAAACCAGGC AAGCCATAGG
RNASE1	2B	Anneal 62, 35 cycles	CAGTCTGTGAT GTGCATGCTG	CCTGATACTG CTGGTGCTG
RNASE1	2C	Anneal 62, 35 cycles	CTGTGGAGAGG ATGAGGTTGAG	AAAGGTCAC CTGCAAGAACG

(*Continued*)

Gene	Exon	PCR conditions	Primer 1	Primer 2
SF3B1	14	TDOWN65–55, 45 cycles	CCAACTCATGAC TGTCCTTTC	TGAGTCCAGT CTGGGCAAC
SF3B1	15–16	TDOWN65–55, 45 cycles	TGTTGGGGCATA GTTAAAACC	AGTAGTTGGC ATATTCTGCATCC
USP9X	2	TDOWN65–55, 45 cycles	CTGACAAATGCT GGTACTTCATC	ACTGAGTCCA GCCCCACTG
USP9X	3	Anneal 62, 35 cycles	TGCAATGCTTGT CTATGTTGG	CCTGTCTGCTCTCTTTCTTGG
USP9X	4	Anneal 62, 35 cycles	GCTCATTTGTAGT GCCTCTTTTAG	ATCCACTTC ATCCGCTTTTG
USP9X	5	Anneal 62, 35 cycles	TTGGACAATGTA AAAACAACCAG	TCTTCCCAC TCACAGGCATC
USP9X	6	Anneal 59.5, 35 + 2.75 mM MgCl2	TGTTTGAAATTG CAGTGTTTTG	TTGTCACAC TTTAAATACAGATCACTG
USP9X	7	TDOWN65–55, 45	CGTGCTTTTTACC CTTTAAAGTAGG	GCAAAAAC CAGCAATTCTACG
USP9X	8	Anneal 59.5, 35 + 2.75 mM MgCl2	TTTTTCAAGTGTG GTTTCTTCAAC	GTATGATTC TAAAATCGGCATTAAC
USP9X	9	TDOWN65–55, 45 cycles	TTGAAATAATTG TTAATGCCGATTT	GCTAGAGC TATTCTAATTTCTTGTCTT
USP9X	10	TDOWN65–55, 45 + 2.75 mM MgCl2	AAAAATTGTTTTA ATTTCTGCTTTC	CAGCTACTG TATTTCTTCAGTTTTTAG
USP9X	11	TDOWN65–55, 45 cycles	CTTTATTAATTGTC AGCAAGCAGT	TTGGCTAAA AGTAATTCACAGATCTAA
USP9X	12	TDOWN65–55, 45 cycles	TGGTGACTAAATA TGTGTTGTTTTC	GGGGAAAA GTCACAAAAAGTG
USP9X	13	TDOWN65–55, 45 cycles	AATTTGAAAATAA AATTCTCCTGAGT	GCCAGCCA ATATGCTGTTTC
USP9X	14	TDOWN65–55, 45 cycles	TTCTGGTTACAACA GAGTTTAATTGC	GGTCCCTGC TATTTCCCATAG
USP9X	15	TDOWN65–55, 45 cycles	TGATTTGGGTTATC CATGTG	ACTTGGGGA CTTGGGAAAAG
USP9X	16	Anneal 59.5, 35 + 2.75 mM MgCl2	TGCCTTTCCGGAT AAAAATTAC	CAATTCCTTA CGTACATGAAAAAG
USP9X	17	TDOWN65–55, 45 cycles	TGTGTTTAAGATA ACTGAGGATATGG	AGAAATGGG GGCCGAAAG
USP9X	18	Anneal 59.5, 35 + 2.75 mM MgCl2	TTAAATCAGCCA TTGCCATC	TCCTTTTACC AAATGGCTAGG
USP9X	19	Anneal 59.5, 35 + 2.75 mM MgCl2	CCCCAGCATTAT TTTCTTGG	TTTACTGATTA AACGATAGTCTCTGG
USP9X	20	TDOWN65–55, 45 cycles	TGTGTAAGTATT TTTCCTTGTTTTTG	CCTAGCATGA TGCCTTACATAC

(*Continued*)

Gene	Exon	PCR conditions	Primer 1	Primer 2
USP9X	21	Anneal 59.5, 35 + 2.75 mM MgCl2	CAGAGAAACTTA CTTGGGCTTTT	ACTCCTTTAT TGTCCAGCAAGT
USP9X	22	TDOWN65–55, 45 cycles	TGGTCTTCAAGA TATCAAAAGTTAAA	AAAAGGAAA AACAAAGAAAACAAC
USP9X	23	Anneal 62, 35 cycles	TCAAGAGTTTCT TGGCACTGAC	TTGAGTCAA CCTTGAACTCTACAC
USP9X	24	TDOWN65–55, 45 cycles	AAGAGCAATTG GTTAGGTCTGG	AAATAAGTG GGCCTAGGAAAG
USP9X	25	TDOWN65–55, 45 cycles	TTTCTAAACATA ATTTCACTCTCATTC	CAAACCACA TGCACAATTAAG
USP9X	26	TDOWN65–55, 45 cycles	TTTTTCTTAAATA CTTCTTTGGGTAAT	GCCTAAATTA ATTCACTCCATTTACA
USP9X	27	TDOWN65–55, 45 cycles	CACCTCTCCTC CCCAAGAG	TTGCTTAAA GAATGAATCACATTG
USP9X	28	Anneal 64, 35 + 2.75 mM MgCl2	GGATTTTGAGG ATGGACGTG	TTTTCCATTT AACCAAAATGCTC
USP9X	29	Anneal 62, 35 cycles	CTGGCTCCTCT CTCCCTCTT	GGTTATGATA TGTAGCTTACAACCAAG
USP9X	30	TDOWN65–55, 45 + 2.75 mM MgCl2	GCATTGCTAATA TGTAATCCCTTTT	TGAAATGAT CAATTATCACATTCTATT
USP9X	31	TDOWN65–55, 45 cycles	AATGGCCGTGT TCATGCTAC	TGAAACACA GAAAACAGAAATGG
USP9X	32	TDOWN65–55, 45 cycles	TTTAGCTCATT GGTGACATTTTT	TGTATTGAG ATGTTCATTTTAAGAAGT
USP9X	33	Anneal 59.5, 35 + 2.75 mM MgCl2	TTGTACATATTGTT CTGTGAATGTTAC	CATGGTACA ACATATACTTTCAATGTG
USP9X	34	TDOWN65–55, 45 cycles	AAAATGTTTACAAA ACTAAGGGATAAA	AGGGTGAC TTGTGAGGATGAA
USP9X	35a	Anneal 59.5, 35 + 2.75 mM MgCl2	TCCTGTGGATTTTA ATAGAACATCTG	ACTCTCCA CCAAAACACTGG
USP9X	35b	Anneal 59.5, 35 + 2.75 mM MgCl2	AAGGAATGGTGG AGATGGTG	CCACACCC AGCAAATTAGAC
USP9X	36	TDOWN65–55, 45 cycles	TTGTTTGAAAAT GGGGTTGG	TTAAAGAA GCAAACCAAACATTAG
USP9X	37a	Anneal 64, 35 + 2.75 mM MgCl2	GCTGGTTCTCT CCAGCAGTA	CTTCTGCA CTAGGGCACTCC
USP9X	37b	Anneal 62, 35 cycles	TTTTTGGTTTGC TCATAACGTC	CTTCGACTT CCAGGAAACAC
USP9X	38	TDOWN65–55, 45 cycles	TGCATGTCCTTT TTATGCAAG	TTGACCTAA ATGACTTCACAATTT
USP9X	39	Anneal 64, 35 cycles	TTTTCTCCCTCT CTTTCTTCTCC	GCAAAACT CTGTCTCAAAACAAAC
USP9X	40	TDOWN65–55, 45 cycles	GCTATTTTCTATC GTGAAAGTTGTG	GACATGGT GAGTAACTTAATGAAAGG

(*Continued*)

Gene	Exon	PCR conditions	Primer 1	Primer 2
USP9X	41	TDOWN65–55, 45 cycles	CTTCTCTGGCA GGTAAAAGG	ACTCTAACA CCTGGGCAAGC
USP9X	42	TDOWN65–55, 45 + 2.75 mM MgCl2	TTTTATTTTATATC TGGTTCTCTTTCA	TGACAACC CCAAGTTCTCAT
USP9X	43	TDOWN65–55, 45 cycles	TCATGAGAACTT GGGGTTGTC	TTTTCAAAA CCCAAAACTTCAAC
USP9X	44	TDOWN65–55, 45 cycles	AAAAGTAAACTT GAGTTTGCCTACG	CAACAAACA ATTGAGCCATCC
USP9X	45	TDOWN65–55, 45 cycles	CCATGATTTTGAA GTTGTTTTACTATG	CAGAAAAAG GTTGGACTGTAAGG
